# Does restrictive anorexia nervosa impact brain aging? A machine learning approach to estimate age based on brain structure

**DOI:** 10.1016/j.compbiomed.2025.110484

**Published:** 2025-06-13

**Authors:** Yubraj Gupta, Feliberto de la Cruz, Katrin Rieger, Monica di Giuliano, Christian Gaser, James Cole, Lauren Breithaupt, Laura M. Holsen, Kamryn T. Eddy, Jennifer J. Thomas, Suheyla Cetin-Karayumak, Marek Kubicki, Elizabeth A. Lawson, Karen K. Miller, Madhusmita Misra, Andy Schumann, Karl-Jürgen Bär

**Affiliations:** a Department of Psychosomatic Medicine and Psychotherapy, Jena University Hospital, Jena, Germany; b Department of Psychiatry, Brigham and Women’s Hospital, Harvard Medical School, Boston, USA; c Department of Psychiatry and Psychotherapy, Jena University Hospital, Jena, Germany; d Department of Neurology, Jena University Hospital, Jena, Germany; e Centre for Medical Imaging Computer, University College London, London, UK; f Dementia Research Centre, University College London, London, UK; g Eating Disorders Clinical and Research Program, Massachusetts General Hospital, Boston, USA; h Department of Psychiatry, Harvard Medical School, Boston, USA; i Mass General Brigham Multidisciplinary Eating Disorder Research Collaborative, Massachusetts General Hospital, Boston, USA; j Athinoula A. Martinos Center, Massachusetts General Hospital, Boston, USA; k Harvard Medical School, Boston, USA; l Neuroendocrine Unit, Massachusetts General Hospital, Boston, USA; m Division of Pediatric Endocrinology, University of Virginia, Charlottesville, USA; n Department of Pediatrics, University of Virginia, Charlottesville, USA; o German Center for Mental Health (DZPG), Germany

**Keywords:** Eating disorder, Acute anorexia nervosa (acAN), Weight-restored AN (wrAN), Brain aging, Structural MRI, Machine learning (ML), Brain-PAD

## Abstract

Anorexia nervosa (AN), a severe eating disorder marked by extreme weight loss and malnutrition, leads to significant alterations in brain structure. This study used machine learning (ML) to estimate brain age from structural MRI scans and investigated brain-predicted age difference (brain-PAD) as a potential biomarker in AN. Structural MRI scans were collected from female participants aged 10–40 years across two institutions (Boston, USA, and Jena, Germany), including acute AN (acAN; *n*=113), weight-restored AN (wrAN; *n*=35), and age-matched healthy controls (HC; *n*=90). The ML model was trained on 3487 healthy female participants (ages 5–45 years) from ten datasets, using 377 neuroanatomical features extracted from T1-weighted MRI scans.

The model achieved strong performance with a mean absolute error (MAE) of 1.93 years and a correlation of *r* = 0.88 in HCs. In acAN patients, brain age was overestimated by an average of +2.25 years, suggesting advanced brain aging. In contrast, wrAN participants showed significantly lower brain-PAD than acAN (+0.26 years, *p*=0.0026) and did not differ from HC (*p*=0.98), suggesting normalization of brain age estimates following weight restoration. A significant group-by-age interaction effect on predicted brain age (*p* < 0.001) indicated that brain age deviations were most pronounced in younger acAN participants. Brain-PAD in acAN was significantly negatively associated with BMI (*r* = −0.291, *p*_fdr_ = 0.005), but not in wrAN or HC groups. Importantly, no significant associations were found between brain-PAD and clinical symptom severity.

These findings suggest that acute AN is linked to advanced brain aging during the acute stage, and that may partially normalize following weight recovery.

## 1. Introduction

Anorexia nervosa (AN) is a severe psychiatric eating disorder characterized by an intense fear of gaining weight and a distorted body image, leading to restricted food intake, malnutrition, and excessive weight loss [1]. Patients with AN commonly limit their caloric intake, often avoiding calorie-dense foods and demonstrating irregular eating behaviors such as prolonged mealtime duration and narrow food preferences [2–4]. Studies have shown that even before diagnosis, individuals at risk for AN tend to consume significantly fewer calories than their healthy peers [5]. This restrictive dietary pattern often includes a preference for vegetarianism, further limiting essential nutrients like protein and certain amino acids, which are vital for healthy body function [2,6–8]. These disordered eating behaviors not only contribute to severe malnutrition but also exacerbate the psychological and physical complications associated with the disorder.

Moreover, AN holds the grim distinction of having one of the highest mortality rates among psychiatric disorders [9], with more than 5% of patients dying within four years of initial diagnosis [10–12]. Notably, about half of these deaths are due to suicide, while the rest are often due to medical complications arising from the disorder, such as sudden cardiac arrest [1,11,13,14]. Although AN is observed globally, its prevalence is notably higher in developed countries and primarily affects adolescents and young females [1].

Understanding the neurological underpinnings of patients with acute AN (acAN) is essential, considering the disorder typically arises during a critical period of brain development [15,16] and can have long-lasting cognitive [17], emotional, and behavioral consequences [18]. Malnutrition disrupts crucial brain maturation processes, such as myelinogenesis and dendritic pruning, which rely on dietary precursors such as polyunsaturated fatty acids [19–21].

Several studies have investigated the structural brain changes in individuals with acAN compared to healthy control (HC) [22–28], finding significant reductions in the volume of white matter (WM) [29,30] and gray matter (GM) [25,27,28,30] in various brain regions. These reductions in WM and GM are most prominent in brain regions with crucial roles for motor, cognitive, and emotional functions, including the bilateral cerebellum, middle and posterior cingulate gyrus, supplementary motor cortex, precentral gyrus medial segment, hippocampus, and thalamus [22,31,32]. Additionally, individuals with acAN exhibit alterations of a wide range of GM structural properties, such as surface area [19,27,33], curvature [24,34,35], thickness [23,26,27,36,37], and volumes [25,26,38]. Most of these changes have been demonstrated to depend on the BMI of patients [36,39,40]. These extensive changes suggest a strong link between undernutrition and the structural changes observed in acAN. However, studies also show that in partially or fully weight-restored patients with AN, some structural alterations normalize again, indicating that some degree of reversibility can occur with weight recovery [9,26,27,39–42].

Despite significant insights into structural brain alterations in acAN, one largely unexplored domain is how these changes relate to the natural brain aging and development process. Brain aging is characterized by progressive changes in brain structure and function, including reductions in GM volume and cortical thinning (CT) [43,44]. Research shows that the most rapid CT occurs before age 20, with peak changes observed between ages 10 and 17 [45,46]. This process reflects normative maturation, including synaptic pruning and increased myelination, contributing to more efficient brain function. However, AN frequently emerges during this critical window of neurodevelopment. During puberty, key structural networks such as the frontostriatal motivation system and the social brain undergo significant remodeling [47,48]. We hypothesize that malnutrition during this sensitive period may disrupt or exaggerate normal developmental thinning, leading to deviations from age-typical brain trajectories. When brain age models trained on healthy development are applied, such deviations may appear as advanced brain age, especially if the observed structure resembles that of older individuals. Thus, while cortical thinning is part of normal development, abnormal or premature thinning due to AN may result in a mismatch between chronological and predicted brain age.

Given these structural changes across development, machine learning (ML) has become an increasingly popular tool to quantify agerelated brain differences [49]. Across the lifespan, from adolescence to old age, these models have demonstrated a remarkable ability to estimate the age of healthy individuals with precision by learning from structural brain scans. Moreover, ML models have been effectively utilized in the study of psychiatric and neurodegenerative disorders, demonstrating promising results in identifying deviations from typical patterns of brain aging [50–58]. Considering the success of ML brain aging models in conditions such as schizophrenia and Alzheimer’s disease, this approach could also be beneficial in studying AN, potentially revealing either accelerations or delays in brain aging processes.

ML-based brain aging models have revealed that psychiatric and neurodegenerative conditions may exhibit signs of advanced brain aging [59–65], allowing for comparisons between an individual’s brain structure and normative references. For instance, brain age models have detected advanced brain aging in patients with Alzheimer’s disease by up to seven years [62,63]. Even individuals with progressive mild cognitive impairment, which may be a precursor to Alzheimer’s disease, show advanced brain age compared to those with stable mild cognitive impairment [60,64]. In mental disorders like schizophrenia and major depressive disorder, advanced brain aging has been associated with cognitive deficits, symptom severity, and treatment responses [59,61, 66]. These findings highlight the potential of brain aging models to offer valuable insights into the neurobiological mechanisms underlying various psychiatric conditions.

Exploring brain aging as a biomarker for the development and progression of AN could have a significant impact, particularly in relation to cognitive impairments and susceptibility to neurodegenerative diseases later in life. As outlined above, we hypothesize that this brain age discrepancy in AN may stem from disrupted or exaggerated cortical thinning during adolescence, a period of rapid neurodevelopment. This study applies machine learning–based brain age modeling to test whether such disruptions are reflected in advanced brain-PAD, and how they relate to clinical outcomes. The first study investigating brain age in AN provided initial evidence of accelerated aging [67]. However, their model was trained on a relatively small dataset (n = 226) with a limited age range of 5–23 years and relied on relevance vector regression (RVR), a method that may lack the flexibility to capture complex, nonlinear developmental trajectories. These methodological limitations underscore the need for larger, more representative samples and advanced modeling techniques. Given that AN profoundly affects nutritional status [68,69] and cognitive-emotional processes [17,70–73], we hypothesize that these disturbances are linked to altered brain development and advanced brain age.

This study extracted 377 cortical and subcortical features from each structural MRI scan to estimate brain age. We leveraged support vector regression (SVR) and deep kernel learning (DKL)-based Gaussian process regression (GPR), given their ability to capture complex, non-linear relationships in high-dimensional neuroimaging data. The predictive models were trained and validated exclusively on a normative dataset of 3487 healthy female participants aged 5–45 years. By learning how brain structure typically changes with age, the models established a normative aging trajectory. This allowed us to detect deviations in brain development in clinical groups by comparing predicted brain age to chronological age using brain-PAD. Model performance was evaluated using standard metrics such as mean absolute error (MAE), root mean squared error (RMSE), and Pearson correlation coefficients, all computed on held-out validation data to ensure generalizability. The final model was then applied to independent test datasets comprising acAN, wrAN, and HC participants to assess group-level deviations from normative brain aging.

In addition to brain age prediction, we also performed group comparison analysis across the same 377 features to examine structural differences between acAN, wrAN, and HC participants. This secondary analysis examined regional differences in cortical volume, thickness, surface area, mean curvature, and white matter volume across groups. Cohen’s *d* effect sizes were computed to quantify the magnitude of these differences and explore whether weight restoration influences structural recovery in AN.

By estimating the brain-predicted age difference (brain-PAD) relative to chronological age, we aimed to quantify deviations from typical brain aging in our test data, including AN patients and HCs. Given the consistent evidence of premature or excessive cortical thinning in AN patients, we hypothesized that this group would exhibit older brain age estimates and a positive brain-PAD. Additionally, we investigated the relationship between brain-PAD and various clinical profiles of AN patients to examine potential associations between altered brain aging and the clinical characteristics of AN.

## 2. Materials and methods

### 2.1. Participants

**Fig. 1.**
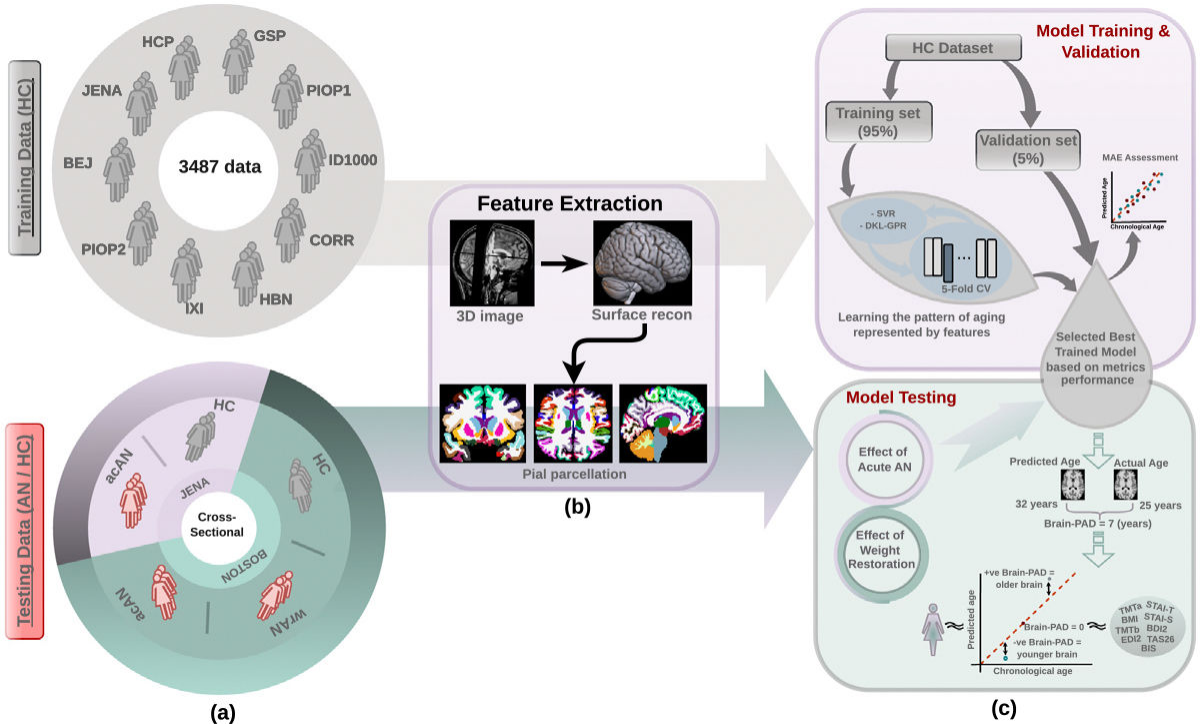
Overview of the ML Pipeline for Brain Age Prediction in AN Patients, (a) Training and testing datasets: The training dataset consisted of 3487 HC samples from 10 datasets (aged 5–45). Testing data from the age group between 10 and 40 includes 42 acAN from Jena University Hospital (JUH), 71 acAN patients from Massachusetts General Hospital (MGH), 35 wrAN from MGH, and age-matched 42 and 48 HC samples from JUH and MGH. (b) Feature extraction using FreeSurfer: Structural MRI scans from HC and AN subjects were processed using FreeSurfer to extract 377 cortical and subcortical features. These features served as inputs to the ML models. (c) Model training and validation: The ML models, including SVR and DKL-GPR, were trained on 95% of the HC data with a 5-fold cross-validation approach to tune hyperparameters. The remaining 5% of HC data was used as a validation set. Model performance was evaluated based on MAE, MSE, RMSE, and correlation coefficient between predicted and chronological age. Model testing and brain-PAD calculation: The best-trained models were tested on the AN and HC testing datasets. Brain-PAD was computed as the difference between the predicted and chronological brain age. Positive brain-PAD values indicate advanced brain aging, while negative values suggest delayed aging. Brain-PAD was correlated with clinical scales, including BMI, TMT-A, TMT-B, STAI-T, STAI-S, BDI-II, TAS-26, EDI-2, and BIS, to assess the relationship between brain aging and clinical symptoms in AN patients.

The datasets utilized in this study included both healthy controls (HC) and participants with anorexia nervosa (AN), focusing exclusively on preadolescent and young adult females aged 5–45 years (see Fig. 1). To build a robust brain-age prediction model capable of capturing normative age-related neuroanatomical variation, we compiled a large dataset of 3487 healthy female participants–3108 sourced from nine publicly available databases and 379 obtained directly from Jena University Hospital (JUH). Detailed information about these data sources is provided in the supplementary material file 1.

Each publicly available database received ethical approval, and informed consent was obtained under local guidelines at each site. The clinical samples were acquired from JUH and MGH to test our model.

#### 2.1.1. Demographics and data acquisition at JUH

For the JUH cohort, 42 individuals diagnosed with the restrictive subtype of acAN (ages 18–40 years; mean age 23.5 ±5.2 years) were included. All patients met DSM-5 criteria for AN according to the Structured Clinical Interview for DSM-5 Axis I disorders [74]. Body mass index (BMI, kg/m^2^) was measured on the day of scanning, along with general psychopathology assessments using standardized tests. These included the Trail Making Test (TMTa and TMTb) [75], Behavioral Inhibition System (BIS) [76], Toronto Alexithymia Scale (TAS26) [77], Eating Disorder Inventory-2 (EDI-2) [78], State-Trait Anxiety Inventory (STAI-Trait, STAI-State) [79], and Beck Depression Inventory (BDI-II) [80]. Additionally, BMI at admission and discharge was documented to evaluate weight changes during treatment. This comprehensive evaluation enabled a multidimensional analysis of the disorder’s impact on brain structure and function.

Data collection began between the third and seventh day after hospital admission to allow patients to acclimate to the hospital routine and minimize intervention interference. All acAN participants were right-handed, as evaluated by the modified version of Annett’s handedness inventory [81]. Similarly, we recruited age-matched HC participants (*n* = 42) who were right-handed and obtained the same assessments as the acAN patients (see Table 1).

Both acquired acAN and HC participants underwent thorough clinical examinations and routine laboratory investigations. They were screened to exclude any history of neurological disorders or psychiatric conditions such as major depression, personality disorders, or obsessive-compulsive disorder. According to the SCID-I interview, none of the HCs (42 for testing and 379 for training data acquired at JUH) had a current episode or a history of mental disorder. For acAN patients, an expert clinician confirmed the absence of comorbid psychiatric diagnoses other than eating disorders.

All participants (and legal guardians for participants under 18) gave informed written consent. The study was approved by the local Ethics Committee of JUH and conducted according to the Declaration of Helsinki (2013). Table 1 provides detailed demographic and clinical information for the HC and AN patient groups.

MRI data were collected using a 3T whole-body system with a 12-element head matrix coil (MAGNETOM Prisma, Siemens Healthcare, Erlangen, Germany). High-resolution anatomical T1-weighted images were acquired using the magnetization-prepared rapid gradient-echo (MPRAGE) sequence in the sagittal plane. Imaging parameters were: repetition time = 2300 ms; echo time = 3.03 ms; inversion time = 900 ms; flip angle = 9°; matrix size = 256 × 256; voxel size = 1 × 1 × 1 mm^3^; number of slices = 192. These parameters were optimized to ensure high-quality structural images suitable for subsequent brain morphometry analysis [82].

#### 2.1.2. Demographics and data acquisition at MGH

This study recruited a total of 71 female participants (ages 10–32 years; mean age 19.9 ±3.9 years) diagnosed with restrictive subtype acute anorexia nervosa, according to DSM-5 criteria, from the New England region. Recruitment was conducted through advertisements, flyers, healthcare providers, outpatient practices, and higher levels of care programs. The exclusion criteria encompassed the use of systemic hormones, pregnancy, history of psychosis, active substance abuse, active suicidal ideation, history of gastrointestinal surgery, or other medical conditions that could result in low body weight, such as neoplasia or diabetes mellitus.

Additionally, the study included 35 weight-recovered female participants with restrictive anorexia nervosa (ages 16–34 years; mean age 22.7 ±3.9 years) (see Table 1). These participants were between 90%–110% of expected body weight for at least six months prior to the study. Weight-recovered AN met DSM-5 diagnostic criteria of restrictive AN in the past. For comparison, a group of healthy women with no lifetime history of any psychiatric diagnoses or eating disorders, with a normal BMI (percentile for age and sex between the 25th and 85th percentile), were scanned (see Table 1).

The studies received approval from the Massachusetts General Hospital Institutional Review Board. Informed consent was obtained from adult participants, and for those under 18, parental/guardian consent and child assent were secured. On the day of MRI acquisition, height (measured in triplicate using a wall-mounted stadiometer) and weight (measured on an electronic scale) were recorded to calculate BMI.

A sagittal 3D T1-weighted MPRAGE sequence was obtained using a Siemens 3 T (3T) Trio scanner (Siemens, Erlangen, Germany), which featured a 12-channel head coil. The imaging parameters included a repetition time (TR) of 2530 ms, an echo time (TE) of 3.43 ms, a flip angle of 7°, and a field of view (FOV) of 256 × 256 mm, with an effective slice thickness of 1.33 mm over 128 slices. To reduce head movement during the scan, foam cushions were used for support.

### 2.2. Neuroimaging processing and feature extraction

After collecting the HC and AN data, we processed each sample using FreeSurfer software version 7.3.2 [83], a widely used tool for analyzing structural MRI data to extract detailed morphological features from cortical and subcortical regions. We utilized the fully automated ‘‘recon-all -all’’ pipeline in FreeSurfer for initial preprocessing, which includes steps to ensure data quality and accuracy, such as motion correction, intensity normalization, transformation into Talairach space, skull stripping, volumetric labeling, segmentation, smoothing, and cortical and subcortical parcellation.

Following preprocessing, we conducted a quality assessment according to FreeSurfer guidelines, checking for errors such as skull stripping inaccuracies, segmentation errors, pial surface misalignments, and topological defects. For participants whose images required correction, we manually edited the data and repeated the necessary preprocessing steps to ensure accuracy. After preprocessing all MRI images, we obtained 377 distinct features using the Desikan-Killiany atlas as a reference. These included five morphological properties, cortical volume, surface area, mean curvature, cortical thickness, and white matter volume, measured across 68 cortical regions (68 × 5 = 340), as well as the volumes of 37 subcortical structures, resulting in a total of 377 cortical and subcortical neuroanatomical features. All regions defined by the Desikan-Killiany atlas were included in the analysis, except for the brainstem, which was excluded due to frequent truncation in structural scans and to maintain consistent measurement reliability across datasets.

### 2.3. Brain age modeling and trajectory deviation analysis

We employed two ML algorithms to predict brain age from neuroimaging data: Support Vector Regression with a Radial Basis Function (RBF) kernel and Deep Kernel Learning Gaussian Process Regression. These algorithms were selected for their ability to model complex, nonlinear relationships in high-dimensional datasets typical of structural MRI scans. All 377 features were normalized by removing the mean and scaling to unit variance to ensure equal feature contribution and minimize bias from larger magnitudes.

#### Model Training and Hyperparameter Tuning:

For each algorithm, we constructed a feature matrix (*n* × *g*), where *n* = 3487 represents the number of samples and *g* = 377 represents the number of neuroanatomical features. The dataset was split into a training set (95%) and a validation set (5%), and a 5-fold cross-validation strategy was used within the training data to evaluate model performance and prevent overfitting. In each iteration, the dataset was partitioned into five subsets, with four subsets used for training and one for testing, ensuring each subset served as the testing set once.

To reduce bias and ensure representativeness, all data splits were stratified based on age bins (in 5-year intervals spanning the 5–45 year range) and dataset of origin. This stratified sampling was applied consistently during both the initial train–validation split and throughout the 5-fold cross-validation process. This ensured that both age distribution and scanner/site variability were balanced across all partitions, minimizing dataset shift and improving generalizability across populations.

- **Support Vector Regression (SVR):** SVR was implemented using the scikit-learn library (version 1.3.2) [84], utilizing the RBF kernel to capture non-linear patterns in the data. Hyperparameters, including the penalty parameter C, kernel coefficient γ, and ϵ, were optimized using ‘‘GridSearchCV’’ with 5-fold cross-validation within the training data of each fold. The optimal hyperparameters were selected based on the lowest mean squared error on validation sets.
- **Deep Kernel Learning Gaussian Process Regression (DKL-GPR):** DKL-GPR combines deep neural networks with Gaussian processes to model complex, non-linear relationships. We implemented DKL-GPR using the GPyTorch library (version 1.11) [85]. The architecture included a fully connected neural network with four hidden layers serving as a feature extractor before Gaussian process regression. The ExactGP function from GPyTorch was used to implement the GPR component. Hyperparameters, including the network architecture parameters, length scale, and variance of the RBF kernel function, were manually tuned within the 5-fold cross-validation framework to balance model complexity and prevent overfitting. The Adam optimizer with a 0.003 learning rate was used during training. Additional implementation and mathematical details are provided in the supplementary material file 2.
- **BrainAgeR:** For comparison, we also evaluate BrainAgeR [53, 86–88], a widely used publicly available brain age prediction tool trained on 3377 healthy (mean age = 40.6 years, SD = 21.4, age range 18–92 years) structural MRI data using Gaussian process regression. We applied this model to our test samples and extracted predicted brain age values.

#### Model Testing and Validation:

After training, the SVR, DKL-GPR, and pre-trained BrainAgeR models were evaluated on the 5% validation set using standard performance metrics: MAE, RMSE, and the Pearson correlation coefficient (*r*) between predicted and chronological age. These metrics were computed prior to any age-bias correction to objectively assess raw model accuracy, in line with prior literature [89,90]. To statistically compare prediction performance across models, we applied a non-parametric bootstrap resampling approach (10,000 iterations) to estimate confidence intervals and compute *p*-values for pairwise differences in MAE. This ensured a robust, distribution-free evaluation of model differences. These metrics were computed to compare the different model prediction accuracies.

#### Brain Age Prediction:

Brain age prediction was performed by applying the trained and validated models to independent test datasets (acAN, wrAN, HC). This enabled the quantification of brain aging patterns and allowed us to explore deviations from normative aging trajectories in clinical populations.

- **Age-Bias Correction:** In addition to performance evaluation, we examined systematic deviations between predicted and chronological age, known as age-bias. To account for this, we applied a standard linear regression-based bias correction procedure [57, 89–91]. Specifically, we fit the following regression model in the validation set:

(1) Y=α*Ω+β

where, Y is the model-predicted brain age, Ω is the chronological age, and α and β are the slope and intercept derived from the validation set. These parameters were then used to correct predicted brain ages in all test datasets using the following formula:

(2) $$Bias\;-\;CorrectedBrainAge\;=\;PredictedBrainAge\;+\;\left[\Omega \;-\;\left(\alpha \;*\;\Omega \;+\;\beta \right)\right]$$

This ensured the corrected Brain-PAD values represented true deviations from the normative aging trajectory, independent of systematic model biases. Corrected brain ages were exclusively used for interpretive analyses, such as group comparisons and clinical associations. Conversely, model performance metrics (MAE, RMSE, r) were reported using uncorrected predictions to avoid artificially inflating model accuracy.

- **Brain-PAD Estimation:** To quantify deviations between estimated brain age and chronological age, we computed the brain-predicted age difference (brain-PAD) as follows:

(3) Brain-PAD=PredictedBrainAge-ChronologicalAge

A positive brain-PAD indicates accelerated brain aging (older brain age relative to chronological age), while a negative brain-PAD suggests a younger brain age compared to the chronological age. Brain-PAD was computed for all participants in testing datasets (acAN, wrAN, HC), and group differences were statistically evaluated using general linear models (GLMs), with chronological age included as a covariate.

To assess the agreement between predicted brain age and chronological age across different models, we applied a Bland–Altman analysis. This method plots the difference between predicted and chronological age against their mean and is commonly used to evaluate systematic bias and limits of agreement between two measurement methods. Bland–Altman plots were generated for all three prediction models.

### 2.4. Statistical analysis

To validate our hypothesis and comprehensively interpret our data, we conducted five distinct statistical analyses:

- **Group Differences in Brain-PAD with Age as a Covariate:** To evaluate group differences in brain-PAD across HC, acAN, and wrAN, we applied a general linear model (GLM) with group as a categorical predictor and chronological age as a covariate (BrainPAD ~ Group + Age). Post-hoc pairwise comparisons among groups were performed using estimated marginal means with Tukey correction for multiple testing. This analysis allowed us to determine whether brain-PAD varied significantly among the three groups while accounting for potential age-related differences. Additionally, to examine whether the predicted brain age follows different age trajectories across groups, we modeled an interaction between chronological age and group using predicted brain age as the dependent variable (Predicted Age ~ Group * Age). This approach allowed us to formally test for group-specific differences in brain maturation patterns and detect whether discrepancies in predicted age vary by age across clinical and control groups.
- **Interaction Analysis Between BMI and Brain-PAD Across Groups:** To explore the relationship between BMI and brain-PAD and how this relationship differs by group, corrected for chronological age, we conducted a GLM including BMI, group, and their interaction (BrainPAD ~ BMI * Group + Age). Significant BMI × Group interaction effects were formally tested, and marginal trend (slope) analyses were performed to estimate and compare the strength and direction of the BMI–Brain-PAD association within each group. Pairwise comparisons of slopes between groups were conducted to determine where significant differences occurred. Additionally, a secondary GLM model (BrainPAD ~ Group + BMI) was fitted to ensure that group differences in brain-PAD remained significant even after adjusting for BMI, isolating the effects of group status from BMI.
- **Relationship Between Brain-PAD and Duration of Illness (JUH site only):** To assess the relationship between brain-PAD and the duration of illness within the acAN group (JUH dataset), a simple linear regression analysis was performed (BrainPAD ~ IllnessDuration). This analysis assessed whether longer illness duration was associated with deviations in predicted brain age.
- **Feature Contribution Analysis Using SHAP Values:** To identify the most influential neuroanatomical features contributing to brain age predictions, we applied the SHapley Additive exPlanation (SHAP) algorithm [92]. SHAP values quantify individual feature contributions to the model’s predictions, enabling us to interpret the relative importance and directionality of these features. Specifically, we implemented a robust subsampling approach:

– For 1000 iterations, we randomly selected 80% of the samples from the healthy validation dataset.

– For each subset, SHAP values were computed using the KernelExplainer algorithm.

– Mean absolute SHAP values across all iterations were aggregated, providing a stable and robust ranking of feature importance.

Positive SHAP values indicate that higher feature values contribute to an older predicted brain age (accelerated brain aging), while negative values indicate that features are associated with a younger predicted brain age (slower or delayed brain aging).

To assess group differences in age-related neuroanatomical trajectories, we modeled normative brain development patterns using generalized additive models (GAMs) with cubic spline smoothers (feature ~ s(Age, bs = ‘‘cs’’) + Group + Group:Age). Initially, normative trajectories were modeled exclusively on HC data to establish baseline age-related trends. Predicted values for all participants were then computed from these models, and individual deviations from these predictions were calculated as residuals (observed minus predicted values). These residuals were standardized into z-scores using the standard deviation of residuals within the HC group. Group-level differences in these deviations and z-scores were evaluated using one-way ANOVAs, with multiple comparisons corrected using the false discovery rate (FDR) method.

- **Group-Level Neuroanatomical Differences:** To explore detailed neuroanatomical differences, we analyzed group-level differences in 377 cortical and subcortical neuroanatomical features between the groups (acAN vs. HC and acAN vs. wrAN) using GLMs, explicitly controlling for chronological age (Feature ~ Group + Age). Cohen’s *d* effect sizes were computed for each neuroanatomical feature to quantify the magnitude of group differences. This comprehensive analysis enabled us to identify specific brain regions exhibiting significant structural differences between clinical groups and healthy controls.

All statistical analyses were performed using R (version 3.6.3). To control for type I error due to multiple comparisons, we applied the Benjamini–Hochberg procedure for false discovery rate (FDR) correction [93], where applicable. Results were considered statistically significant at an adjusted threshold of *p* < 0.05 after FDR correction. Similarly, all analyses involving brain-PAD were conducted using agebias corrected predicted ages to avoid residual age dependency and support valid between-group comparisons.

## 3. Results

### 3.1. Model performance

After evaluating the performance of multiple ML models, we selected the DKL-GPR as the most suitable model for brain age prediction due to its superior performance on both validation and test datasets. The DKL-GPR model achieved a higher correlation coefficient (*r* = 0.87) and a lower MAE = 2.33 years compared to the SVR model, which achieved a correlation of *r* = 0.84 and an MAE of 2.50 years for the validation set. These results are summarized in (see supplementary material file 2) Figure S4a. Due to its improved performance, the DKL-GPR model was chosen for further analysis and applied to independent test datasets to estimate brain age in acAN, wrAN, and HC groups.

In the age-matched HC test set, the model demonstrated high predictive accuracy, achieving a correlation of *r* = 0.88 and an MAE of 1.93 years. To evaluate residual age-dependency after bias-correction, we examined the association between brain-PAD and chronological age in the HC test set. This analysis revealed a non-significant positive correlation (*r* = 0.178, *p* = 0.093), indicating minimal residual age bias (see supplementary material file 2, Figure S3a). To further assess the agreement between predicted and chronological age, a Bland–Altman analysis was conducted. This analysis revealed minimal systematic bias (+0.066 years) and narrow limits of agreement (within ±1.96 standard deviation limits (−5.79 to +5.92 years)), suggesting that the DKL-GPR model produces both accurate and consistent brain age estimates in healthy individuals (supplementary Figure S4b).

Furthermore, to ensure the robustness of the model across acquisition sites, we also examined the DKL-GPR model’s performance separately on HC participants from the JUH and MGH datasets. Although minor differences in MAE were observed (JUH: 2.05 years; MGH: 1.82 years), no substantial variation in accuracy was found, supporting the model’s robustness across acquisition settings (see supplementary material file 2, Figure S3b).

For further benchmarking, we applied the BrainAgeR model to the same validation sample. This model yielded a lower correlation (*r* = 0.74) and higher MAE (3.24 years), indicating reduced prediction accuracy relative to DKL-GPR. A Bland–Altman analysis of BrainAgeR showed greater bias and wider limits of agreement (+1.84 years, ranging from −6.99 to +10.67 years), consistent with its performance metrics. These discrepancies likely reflect differences in model architecture, training sample characteristics, and age range, compared to our model, which was trained on a younger sample spanning 5–45 years.

For completeness, we also examined the SVR model’s agreement profile. Although SVR showed nearly negligible bias (−0.0039 years), its limits of agreement (−6.35 to +6.35 years) were wider than those of DKL-GPR. This suggests that while SVR produces unbiased predictions on average, it is less precise than DKL-GPR.

To statistically compare prediction errors between models, we conducted pairwise bootstrapped comparisons of MAEs across 10,000 resamples. The results revealed that DKL-GPR significantly outperformed BrainAgeR (mean MAE difference = −0.91 years, 95% CI: [−1.52, −0.32], *p* = 0.0024) and SVR also showed lower MAE than BrainAgeR (−0.75 years, 95% CI: [−1.36, –0.15], *p* = 0.0154). However, no significant difference was found between DKL-GPR and SVR (*p* = 0.13), supporting that while both non-linear models are strong candidates, DKL-GPR offers marginally better consistency and agreement.

### 3.2. Brain-PAD is increased in acute AN patients

**Fig. 2.**
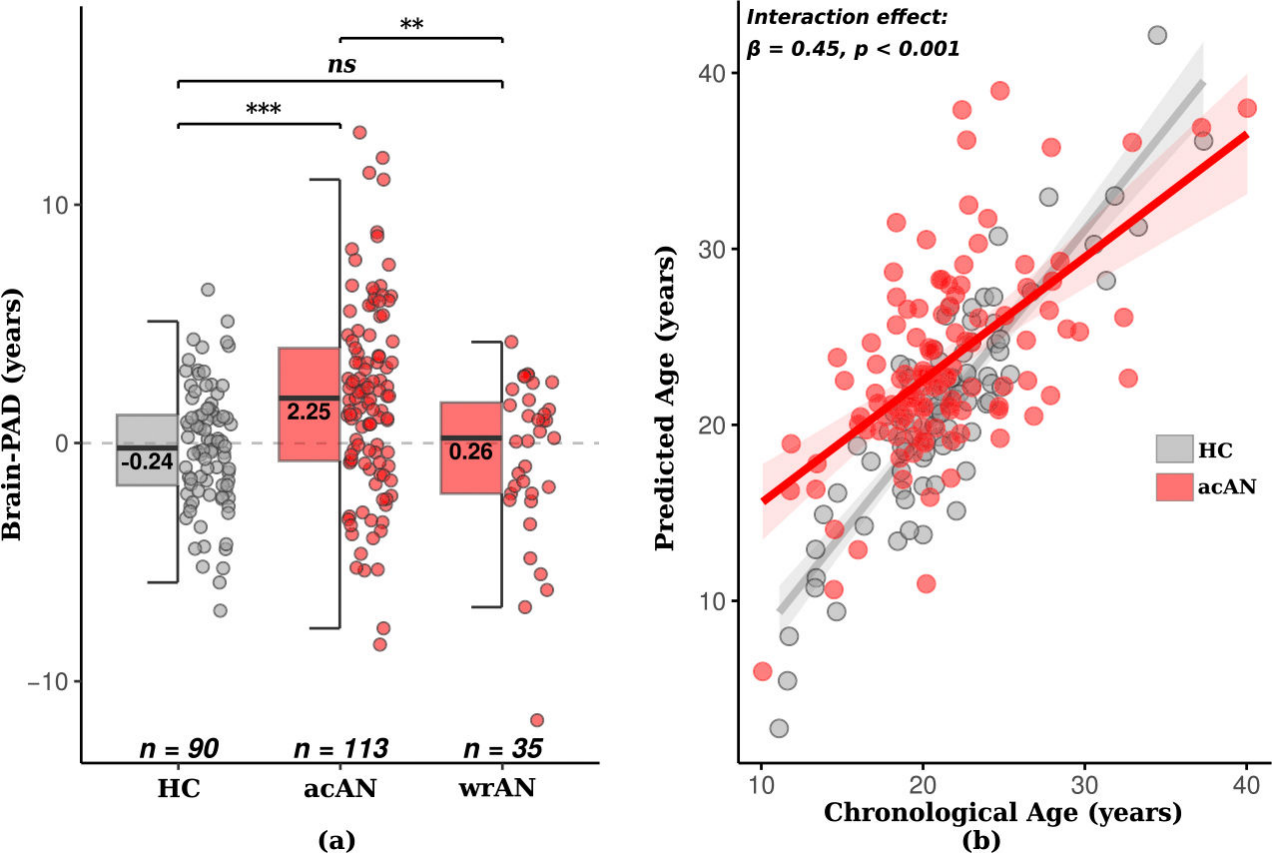
Brain-PAD and Predicted vs. Chronological Age in acAN, wrAN, and Age-matched HC Testing Subjects. (a) Boxplot of brain-PAD values for HC, acAN, and wrAN groups. HC shows a near-zero brain-PAD (median = −0.24 years), acAN patients exhibit significantly higher brain-PAD (median = +2.25 years, *p* < 0.001), and wrAN patients show a reduced brain-PAD (median = +0.26 years). Significant differences are observed between acAN and wrAN (*p* = 0.00261) but not between wrAN and HC (*p* = 0.98). (b) Scatter plot illustrating the relationship between biased-corrected predicted brain age and chronological age for HC and acAN participants. A significant group-by-age interaction effect is observed (*β* = 0.45, *p* < 0.001). Red and gray regression lines represent the fitted trajectories for acAN and HC groups, respectively, with shaded areas indicating 95% confidence intervals. ns = not significant, ** = p-value is less than 0.01, and *** = p-value is less than 0.001.

To formally assess group differences in brain aging, we performed a GLM analysis with group (acAN, wrAN, HC) as the independent variable and chronological age as a covariate. This analysis revealed a significant group effect on brain-PAD. Post-hoc pairwise comparisons indicated that brain-PAD was significantly elevated in the acAN group compared to both HC (*β* = 2.40, *p* < 0.001) and wrAN (*β* = 2.61, *p* = 0.0024). In contrast, no significant difference was observed between wrAN and HC (*p* = 0.96). These findings confirm that elevated brain-PAD is specifically associated with the acute phase of AN and suggest that brain aging tends to normalize following weight restoration (Fig. 2a).

Group-level comparisons of median brain-PAD further support these statistical findings. The HC group (*n* = 90) displayed a near-zero median brain-PAD (−0.24 years), consistent with the model’s accurate estimation of brain age in healthy individuals. Conversely, the acAN group (*n* = 113) showed a significantly elevated median brain-PAD of +2.25 years (*p* < 0.001), reinforcing the presence of advanced brain aging during the acute stage of AN. The wrAN group (*n* = 35) demonstrated a median brain-PAD of +0.26 years, significantly lower than acAN (*p* = 0.00261), but not significantly different from HC (*p* = 0.98).

In addition to brain-PAD group differences, we tested whether the relationship between predicted brain (bias-corrected) age and chronological age varied by group using a separate GLM with an interaction term (Group × Age). This analysis revealed a significant group-by-age interaction between acAN and HC (*β* = 0.45, *p* < 0.001) and between acAN and wrAN (*β* = −0.57, *p* = 0.0018). As shown in Fig. 2b, this indicates that the discrepancy in predicted brain age increases with chronological age, particularly among younger acAN participants, suggesting more pronounced deviations from normative brain aging trajectories in this subgroup.

Importantly, this elevated brain-PAD in acAN patients was consistently replicated across datasets from both institutes (JUH and MGH), underscoring the robustness and reliability of these results across independent samples (see supplementary material file 2, Figure S3b, S3c, and S3d).

### 3.3. Correlation analysis between brain-PAD and clinical scales

We examined the relationship between brain-PAD and clinical variables across acutely ill AN (acAN), wrAN, and HC participants using correlation analyses and GLMs. Our primary goal was to assess how BMI, illness duration, and clinical symptomatology relate to deviations in predicted brain age.

**Fig. 3.**
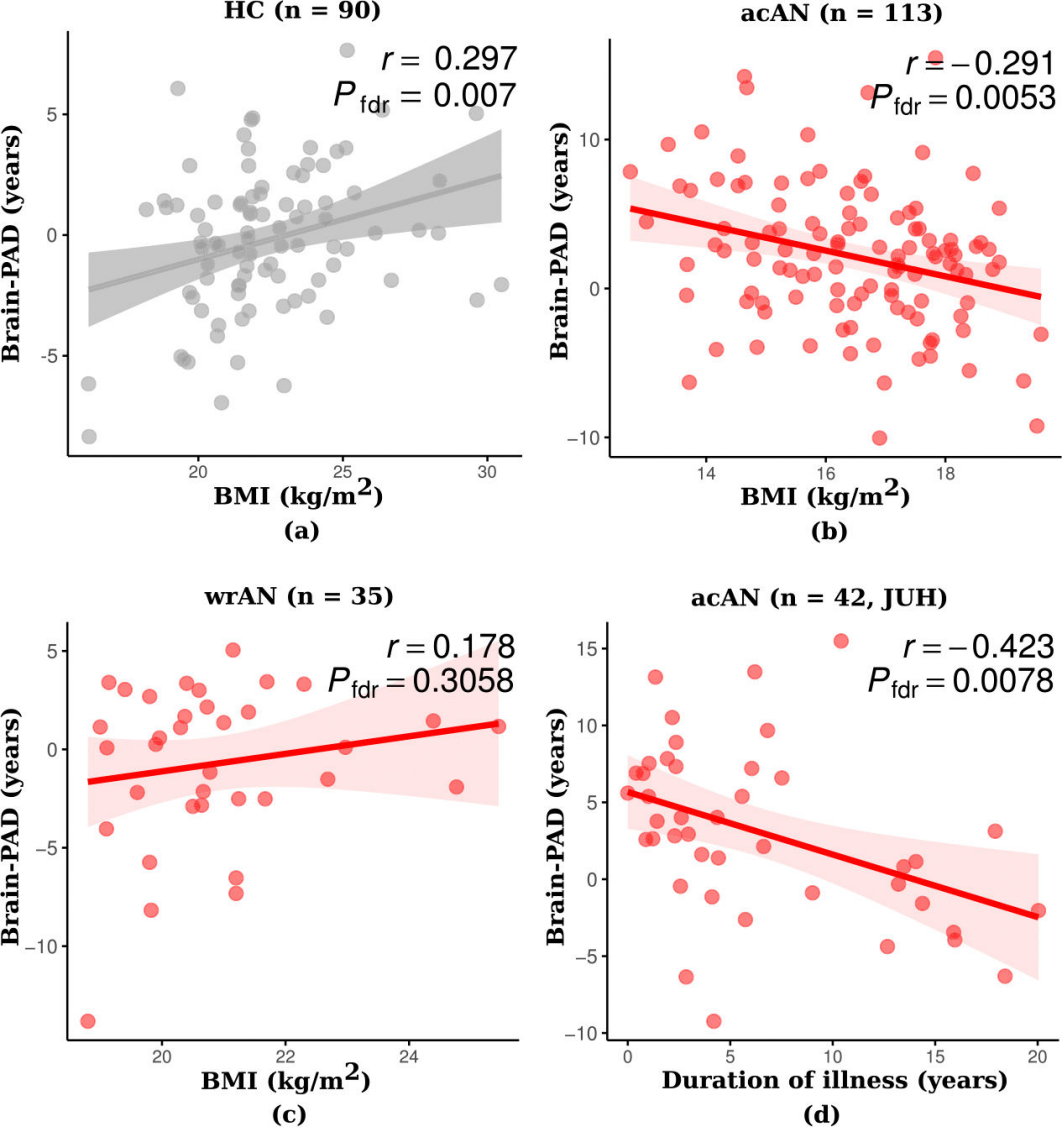
Correlation Analysis Between Brain-PAD with BMI in HC, acAN, and wrAN, and Duration of Illness in acAN in years. (a) Correlation between brain-PAD and BMI in HC samples (*n* = 90), (b) Scatter plot showing a significant negative correlation between brain-PAD and BMI in acAN patients (*n* = 90), with *r* = −0.291 and *p* = 0.0053, indicating that lower BMI was associated with higher brain-PAD values. (c) Correlation between brain-PAD and BMI in wrAN patients (*n* = 35), showing no significant correlation (*r* = 0.178, *p* = 0.3058). (d) A significant negative correlation was found between brain-PAD and the duration of illness in acAN patients, with *r* = −0.423, *p* = 0.0078.

As shown in Fig. 3, BMI was significantly associated with brain-PAD, though the nature of this relationship varied by group. In the acAN group (*n* = 113), a significant negative correlation was observed (*r* = −0.291, *p*_fdr_ = 0.0053), indicating that lower BMI was associated with more advanced brain aging (Fig. 3b). A similar but opposite trend was found in the HC group (*n* = 90), where a positive correlation was observed (*r* = 0.297, *p*_fdr_ = 0.007), suggesting that lower BMI was linked to a younger brain age in healthy individuals (Fig. 3a). In the wrAN group (*n* = 35), no significant correlation was detected (*r* = 0.178, *p* = 0.3058), suggesting partial normalization of brain structure with weight recovery (Fig. 3c).

To formally test whether the association between BMI and brain-PAD differed across groups, we applied a general linear model including BMI, group, chronological age, and their interaction term (Group × BMI). The analysis revealed a significant interaction between BMI and group (*p* < 0.001), indicating that the relationship between BMI and brain-PAD significantly varied by group.

To interpret these differences, we compared the BMI slopes within each group. The BMI–Brain-PAD slope was significantly negative in the acAN group (*β* = −0.823, SE = 0.226, *p* < 0.001), indicating that lower BMI was associated with greater brain age acceleration. In contrast, a positive slope was observed in the HC group (*β* = +0.491, SE = 0.155, *p* < 0.01), suggesting the opposite relationship. The slope in the wrAN group was also positive (*β* = +0.397, SE = 0.407), but not statistically significant (*p* = 0.35). Pairwise comparisons confirmed that the BMI–Brain-PAD slopes differed significantly between acAN and HC (*p* < 0.001) and between acAN and wrAN (*p* = 0.025), while no difference was observed between HC and wrAN (*p* = 0.97).

To ensure that group differences in brain-PAD were not merely driven by BMI, we conducted an additional GLM with group and BMI as predictors of brain-PAD. In this model, group differences remained statistically significant even after adjusting for BMI. Brain-PAD was significantly higher in acAN compared to HC (*β* = 2.43, *p* = 0.0310) and compared to wrAN (*β* = 2.91, *p* = 0.0085), while no significant difference was observed between HC and wrAN (*p* = 0.8369). Importantly, BMI itself was not a significant predictor of brain-PAD (*β* = 0.0054, *p* = 0.966), suggesting that the observed differences are primarily attributable to group status rather than BMI alone. These results further reinforce that advanced brain aging is specifically linked to the acute phase of AN and is not fully explained by differences in body weight.

Additionally, we also examined the relationship between brain-PAD and illness duration (defined as chronological age minus age of onset) in the acAN subgroup from the JUH site (Fig. 3d), where onset age data were available. A significant negative correlation was found (*r* = −0.423, *p* = 0.0078), suggesting that individuals with longer illness durations exhibited lower brain-PAD, potentially reflecting a normalization over time or the effects of treatment exposure.

Finally, we assessed whether brain-PAD was associated with clinical or cognitive symptomatology by correlating brain-PAD with a range of psychological measures. Across the JUH dataset (Table 2), no significant correlations between brain-PAD and any clinical scales survived after FDR correction.

### 3.4. Brain regions associated with aging

**Fig. 4.**
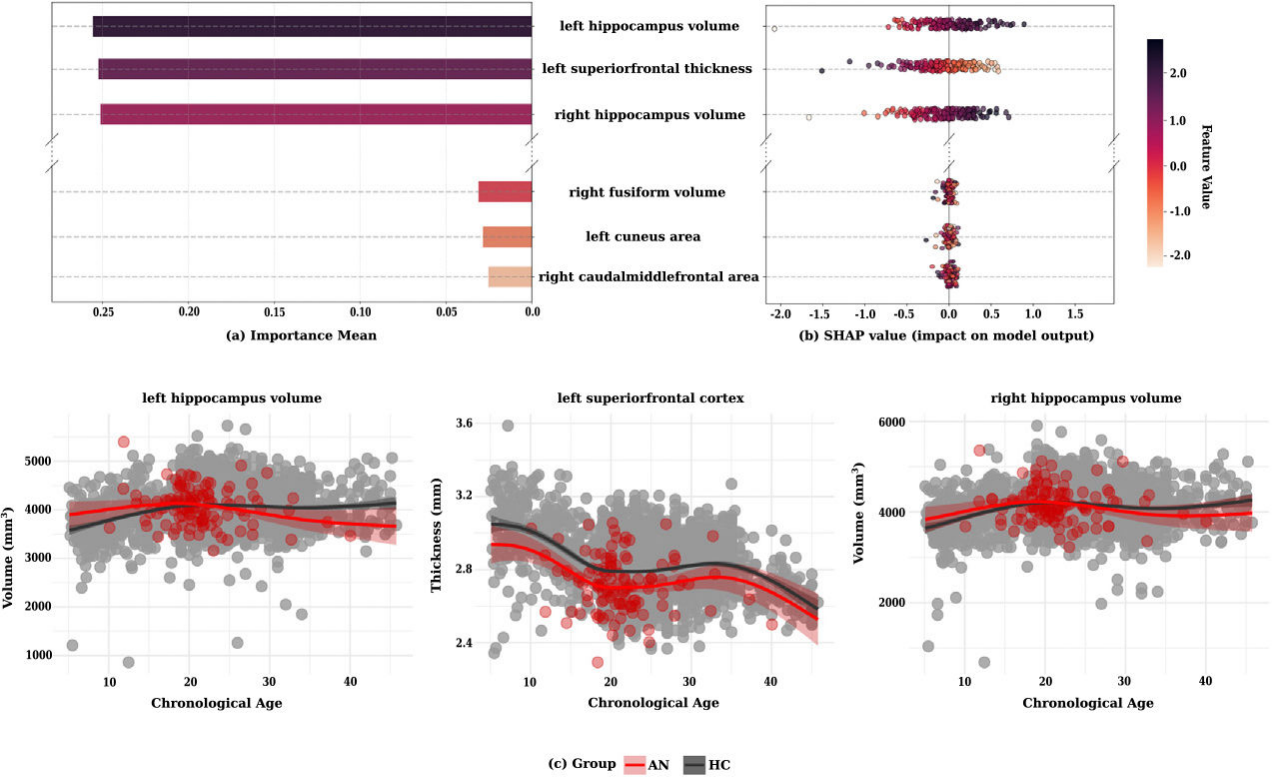
Brain Regions Associated with Aging in AN Patients. (a) The top three and last three brain regions contribute to brain age prediction based on SHAP values from the DKL-GPR model. The left hippocampus volume, left superior frontal cortex thickness, and right hippocampus volume exhibit the highest mean importance, indicating their key roles in brain age prediction for participants with AN. (b) SHAP value distributions for these regions show the direction and magnitude of their impact on brain age predictions. Positive SHAP values are associated with higher predicted brain age (suggesting structural abnormalities), while negative values indicate a younger predicted brain age. (c) Scatter plots of the top three SHAP-selected brain regions (left/right hippocampus and left superior frontal cortex) plotted against chronological age. Smoothing splines (mean ±95% CI) reveal different aging trajectories for acAN patients (red) versus HC (gray), highlighting potential neuroanatomical changes contributing to accelerated aging in AN.

The most significant brain regions for predicting brain age in the HC validation samples were determined through SHAP analysis, which ranked features by their impact on model predictions. The top three features contributing to brain age predictions were the left hippocampal volume, left superior frontal cortex thickness, and right hippocampal volume, with SHAP importance scores of 0.256, 0.252, and 0.251, respectively (Fig. 4a). In contrast, regions such as the right fusiform volume, left cuneus area, and right caudal middle frontal cortex had minimal influence on model predictions.

The SHAP value distributions (Fig. 4b) further illustrate how the structural values of these regions influence brain age predictions. For instance, larger hippocampal volumes are associated with older brain predictions (positive SHAP values), while smaller volumes are linked to younger brain predictions (negative SHAP values). These relationships are consistent with known structural changes across the lifespan, supporting the biological relevance of the brain age model.

To examine whether the aging trajectories of these key features differ between clinical and control groups, we modeled age-feature relationships using GAMs. As shown in Fig. 4c, the hippocampal volumes and superior frontal thickness followed distinct trajectories in acAN (*n* = 113) compared to HC (*n* = 3577, including 90 JUH/MGH HC data), suggesting altered neurodevelopmental patterns in AN. To formally quantify deviation from the normative trajectory, we computed each participant’s residual and z-scored deviation from the HC trajectory. Group comparisons revealed that only the left superior frontal cortex thickness showed a significant deviation in the acAN group relative to HC after FDR correction (*F*(1, 3690) = 46.83, *p*_fdr_ = 2.71e–11). The left and right hippocampal volumes did not show significant group-level deviations (both *p*_fdr_ = 0.923). These findings suggest that while the hippocampus is a strong contributor to model predictions, the superior frontal cortex may be more sensitive to group-level alterations in brain aging trajectories.

### 3.5. Magnitude of individual brain structural features affected in AN patients

**Fig. 5.**
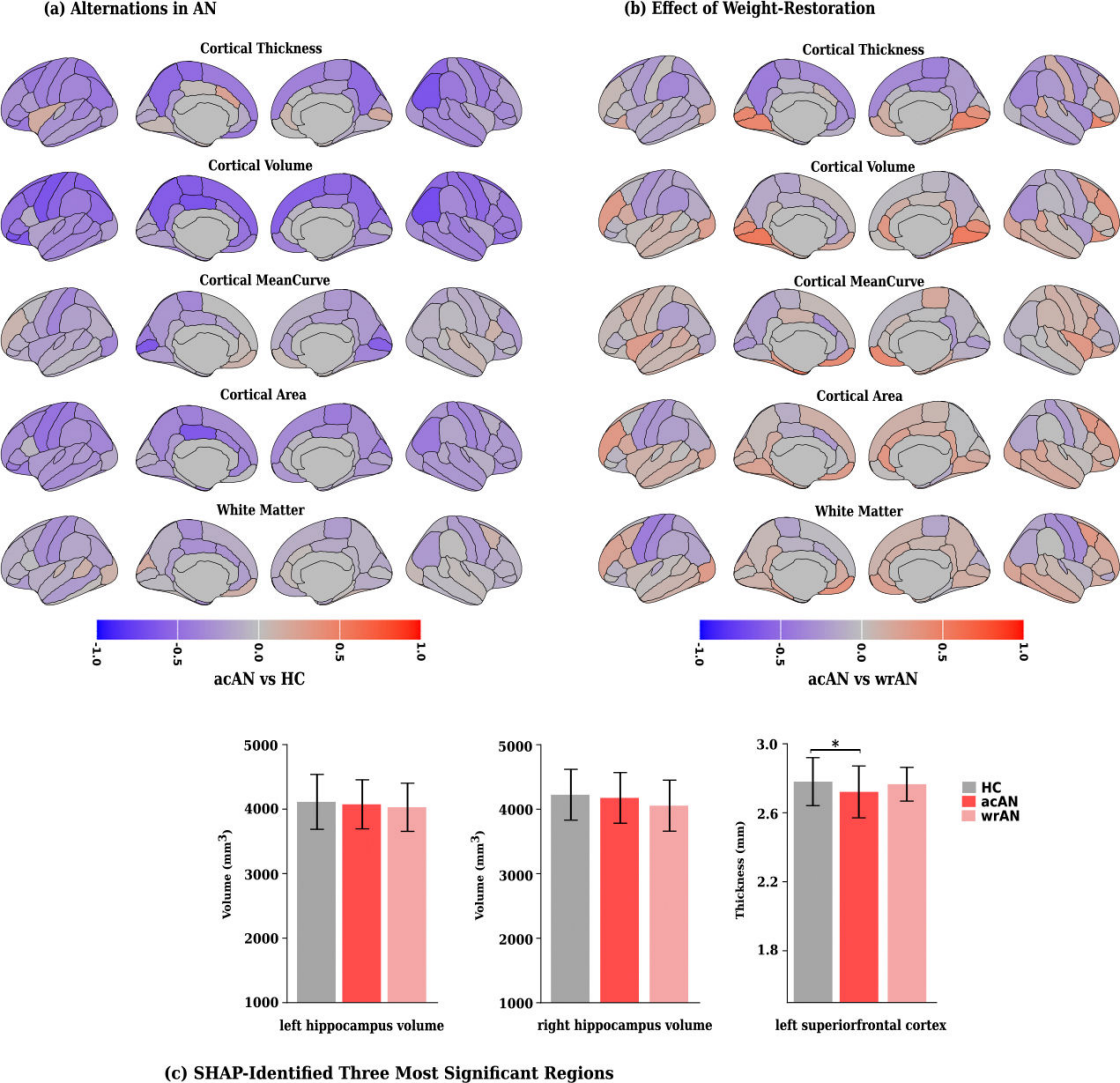
Neuroanatomical Comparisons Between acAN, wrAN, and HC. (a) This panel shows group-level comparisons of cortical and white matter structures between acAN patients and HC participants. The comparisons are displayed across five structural measures: cortical thickness, cortical volume, cortical mean curvature, cortical surface area, and white matter volume. Blue regions represent reductions in acAN relative to HC (negative Cohen’s *d* values), while red regions indicate increases (positive Cohen’s *d* values). (b) Surface-based maps illustrating the effect sizes of structural changes in acAN compared to wrAN patients. Notable reductions in cortical and subcortical structures are seen in acAN, which are partially restored following weight normalization in wrAN. The color bar denotes Cohen’s *d* effect sizes, ranging from −1.0 (blue) to 1.0 (red), with zero (gray) indicating no difference between groups. (c) Bar plots showing the mean volumes of the left hippocampus, right hippocampus, and the mean thickness of the left superior frontal cortex in HC, acAN, and wrAN groups. The left superior frontal thickness shows a significant reduction in acAN compared to HC (*p* = 0.031), while hippocampal volumes show non-significant differences across groups.

Finally, we explored group-level comparisons of all input neuroanatomical features between cohorts to assess structural differences (see Fig. 5 and the supplementary material file 3, Group comparison sheet).

Acute AN participants showed significantly lower cortical and subcortical structural measures compared to HC (see Fig. 5a and supplementary material file 2 Figures S1 and S2). Notably, significant reductions were observed in the right inferior parietal volume (*d* = −0.70, *p*_fdr_ < 0.001) and thickness (*d* = −0.65, *p*_fdr_ < 0.001), and left precentral volume (*d* = −0.65, *p*_fdr_ < 0.001). Conversely, some brain regions showed increases in volume for acAN patients, most pronounced in the third ventricle (*d* = 0.53, *p*_fdr_ = 0.004) and fourth ventricle (*d* = 0.35, *p*_fdr_ = 0.068).

From the most important features revealed by the SHAP-analysis, only the left superior frontal thickness significantly differed between acAN and controls, with patients exhibiting a marked reduction in cortical thickness in this region (*d* = −0.41, *p*_fdr_ = 0.031). Marginal volumetric reductions were observed in the right (*d* = −0.12, n.s.) and left hippocampus (*d* = −0.09, n.s.), which were not statistically significant. In wrAN participants, the superior frontal thickness increased compared to acAN (*d* = −0.32, n.s.), while hippocampal volumes continued to decrease, with the right hippocampus showing a more prominent decrease (*d* = 0.31, n.s.) compared to the left hippocampus (*d* = 0.12, n.s.) (see Fig. 5b and Fig. 5c).

## 4. Discussion

This study investigated deviations in brain aging trajectories among preadolescent and young adult female participants with acute anorexia nervosa (acAN), compared to age-matched HC testing participants. Consistent with our hypothesis, acAN participants exhibited significantly elevated brain-PAD, suggesting accelerated brain aging relative to chronological age. Importantly, lower BMI was associated with greater brain-PAD in the acAN group, implicating malnutrition as a key factor driving these structural brain alterations. Conversely, brain-PAD values in wrAN participants were comparable to those of healthy controls, indicating a potential normalization of brain aging with clinical recovery. To further explore whether weight status alone could explain these group differences, we performed a GLM analysis with group and BMI as predictors of brain-PAD. The results confirmed that group differences in brain-PAD remained significant after adjusting for BMI, whereas BMI itself was not a significant predictor (*p* = 0.966). These findings suggest that structural brain recovery in wrAN may reflect broader neurobiological processes beyond weight restoration alone. Additionally, regions such as the hippocampus and superior frontal cortex, which contributed most strongly to brain age estimation in healthy individuals, also exhibited pronounced structural alterations in acAN participants, underscoring their particular vulnerability to the neurodevelopmental impact of anorexia nervosa.

### 4.1. Validation of the brain age estimation model

Our patient cohort was relatively young (10 to 40 years) compared to samples used in previous brain aging studies, so we designed our brain age prediction model for this developmental period by training it on large normative samples (*n* = 3487) of healthy individuals aged 5–45 years. Five percent of these individuals were randomly selected in 5-year age bins to serve as a validation set, while the remaining 95% were used for training the predictive models, DKL-GPR and SVR. This allowed us to capture the non-linear neurodevelopmental patterns typical of this age group more accurately than existing models trained predominantly on adult samples.

Among the evaluated models, DKL-GPR outperformed both SVR and the widely used BrainAgeR model. DKL-GPR achieved the lowest MAE and highest correlation with chronological age on both the validation (MAE = 2.33, *r* = 0.87) and test datasets (MAE = 1.93, *r* = 0.88 for HC), with minimal residual age bias after correction. In contrast, BrainAgeR, which was trained on adult samples aged 18–92 years, exhibited substantial bias and reduced accuracy in our younger cohort. In general, making direct comparisons of performance metrics (such as MAE, bias) with the BrainAgeR model is inherently limited due to differing age distributions.

To formally assess model differences, we conducted bootstrapped comparisons of MAEs. DKL-GPR showed a statistically significant improvement over BrainAgeR (MAE difference = −0.91 years, 95% CI = [−1.52, −0.32], *p* = 0.0024), and also outperformed SVR, though this comparison did not reach significance (MAE difference = −0.17 years, 95% CI= [-0.3979, 0.0488], *p* = 0.13). Bland–Altman analyses supported these findings, showing tighter agreement and less bias for DKL-GPR compared to other models. These findings align with previous work emphasizing the importance of age-matched training data in brain age modeling [94]. The improved performance of DKL-GPR, both in terms of accuracy and agreement with true age, reinforces its suitability for detecting subtle deviations in neurodevelopment among clinical populations.

### 4.2. Testing brain aging in AN participants

Applying brain age prediction to our clinical cohorts revealed clear evidence of advanced brain aging in acAN. Participants in the acAN group showed significantly elevated brain-PAD values (median = +2.25 years) compared to both HC (median = −0.24 years) and weight-restored AN individuals (wrAN, median = +0.26 years). These group-level differences were confirmed using a GLM controlling for chronological age, where acAN exhibited significantly higher brain-PAD than HC (*p* < 0.001) and wrAN (*p* = 0.0024), while no significant difference was observed between wrAN and HC (*p* = 0.96). This supports the hypothesis that advanced brain aging is specific to the acute underweight phase of AN and tends to normalize following weight restoration.

Additionally, the slope of the regression line between predicted and chronological age was significantly lower in acAN compared to healthy controls, suggesting that brain aging in acAN is not a gradual process but rather appears more accelerated in younger participants, with some normalization in older individuals. This high variability, reflected by the wider spread of data points around the regression line, may be linked to differences in disease severity or illness duration, with more severe cases leading to higher brain-PAD scores. These developmental differences were further supported by a formal group-by-age interaction analysis. A GLM including a group × age interaction term demonstrated a significant interaction effect (groupHC × age: *p* < 0.001; group-wrAN × age: *p* = 0.0018), suggesting that the discrepancy between predicted and chronological age was more pronounced at younger ages among acAN participants. In other words, brain-PAD was highest in younger acAN individuals and gradually decreased with age, eventually converging toward HC values in adulthood. This pattern supports the interpretation that observed brain-PAD elevation in AN is not reflective of a uniform acceleration of aging but may instead result from disrupted developmental trajectories during critical neurodevelopmental windows. One influential factor might be the level of malnutrition, as we observed a significant negative correlation between brain-PAD and BMI (*r* = −0.291, *p*_fdr_ = 0.0053), indicating that lower BMI is associated with more advanced brain aging. Previous studies [23,36, 39] support this finding, showing that reduced cortical volume and thickness in AN are often linked to lower BMI and that severe caloric restriction induces gray matter atrophy, ventricular enlargement, and white matter deficits. Interestingly, this pattern was reversed in the HC group, where a significant positive association between BMI and brain-PAD was observed. In contrast, no significant association was found in the wrAN group. These group-specific effects were further supported by a significant BMI × Group interaction in the GLM, and follow-up analyses revealed that the BMI-Brain-PAD relationship significantly differed between acAN and both HC (*p* < 0.001) and wrAN (*p* = 0.025), but not between HC and wrAN (*p* = 0.97).

We also observed a significant negative correlation between brain-PAD and illness duration (*r* = −0.423, *p* = 0.0078) within the acAN subgroup from the JUH dataset, suggesting that patients with longer illness duration may exhibit a younger predicted brain age relative to chronological age. This finding could indicate that younger patients are more affected by brain structural changes, leading to a higher brain-PAD, or it could reflect some recovery as therapy progresses.

In contrast to acAN, weight-restored participants showed no significant difference in brain-PAD compared to healthy controls, but their brain age still differed from acAN participants, with a median PAD of +0.26 years. Additionally, group comparisons controlling for BMI showed that group effects remained significant between acAN and both HC (*p* = 0.031) and wrAN (*p* = 0.0085), while BMI itself was not a significant predictor (*p* = 0.966). This indicates that normalization of brain aging in wrAN is not merely due to regained weight but may involve broader neurobiological recovery processes. This supports evidence that structural alterations in the brain structure, such as cortical volume, thickness, or gyrification, tend to normalize after partial or full weight recovery in participants with AN [41,95–98]. It is important to note that this observed normalization likely reflects reversible effects of malnutrition, rather than the regeneration of neurons. While structural recovery is evident, it is more likely to result from restoration of hydration, glial cell volume, synaptic density, and remyelination, which are known to be impacted by prolonged caloric restriction. Additionally, recovery of hormonal and metabolic balance (such as cortisol, estrogen, and leptin) may support neuroplasticity and normalization of brain structure. Although true neurogenesis in humans is limited, especially beyond early development, changes in supporting systems, including astrocytic function and vascular integrity, could facilitate tissue recovery. These biological mechanisms may underlie the observed reductions in brain-PAD following weight restoration in wrAN individuals.

However, the persistence of structural abnormalities after weight recovery may reflect the residual effects of long-term malnutrition or indicate a need for further therapeutic interventions to address persistent neurobiological deficits. While partial recovery of brain structure is evident, local alterations still seem to persist to some extent after recovery [99–101]. This residual damage highlights the importance of comprehensive treatments that extend beyond weight restoration and target long-term neurological health.

Although advanced brain aging is evident in acAN, brain-PAD did not correlate significantly with clinical symptom severity (such as mood, anxiety, and cognitive functioning) after correction for multiple comparisons. This observation aligns with previous research [26,27], where structural differences in brain regions like the thalamus and orbitofrontal cortex were observed but were not strongly associated with cognitive or emotional deficits as measured by clinical scales. This suggests that advanced brain aging in AN may be driven more by physiological factors, such as malnutrition, than the psychological symptoms typically measured.

### 4.3. Regional changes contributing to advanced brain age in acans

For our model, the most important features for accurately estimating age in healthy individuals were the hippocampal volume (left and right) and superior frontal cortical thickness, as identified through SHAP analysis. While the hippocampus is well-known for its critical role in early brain development, including memory formation, spatial navigation, and emotional regulation, it remains functionally and structurally relevant throughout the lifespan. Although neurogenesis in the hippocampus is most prominent during childhood, particularly in the dentate gyrus, the structure continues to undergo dynamic changes in adolescence and adulthood, including synaptic remodeling, stress-related atrophy, and age-related volume decline [102,103]. Thus, its importance in brain age prediction likely reflects its continuous plasticity and vulnerability, rather than being restricted to a specific age period. The hippocampus has also been implicated in both neurodevelopmental and neurodegenerative disorders, further underscoring its broad relevance in models spanning the full age range of our study. The hippocampal volume has been shown to expand nonlinearly with increasing age during childhood and adolescence [104–106].

We observed an initial rapid hippocampus growth in healthy individuals that plateaued after age 20, consistent with previous findings of linear growth between ages 5 and 20, followed by a gradual decline [104–106]. In acAN patients, hippocampal growth appeared slower, with a more pronounced decline after age 20. We found marginal reductions in left and right hippocampal volumes in the acAN group compared to HC, even after adjusting for age. These reductions were more pronounced in the wrAN group, indicating further hippocampal volume loss in weight-recovered patients. The hippocampus has been consistently shown to be reduced in volume in AN patients [26,107], potentially contributing to specific cognitive impairments [108]. This hippocampal volume reduction appears not to fully normalize after weight recovery [31,109], which might link to certain cognitive domains, such as memory and learning, being impaired in wrAN [110,111]. A recent study found that structural alterations and their recovery after weight-restoration depend on the subfield of the hippocampus [112].

However, these volumetric reductions in the hippocampus do not lead to overestimating brain age in our model. Since hippocampal volume tends to increase during normal aging, as our healthy training data shows (see Fig. 4c), the model associates larger volumes with an older brain, reflected by a positive SHAP value. Thus, the observed hippocampal alterations in acAN do not appear to contribute to a positive brain-PAD in these patients. Instead, other structural changes likely drive the age overestimation in this group.

Throughout maturation, the brain undergoes significant remodeling, with changes continuing into the mid-twenties to early thirties [113–115]. During this period, cortical thickness decreases as white matter expands to form neural circuits and enhance brain connectivity. These developmental trajectories vary by region, with cortical thinning in the middle frontal cortex following a cubic pattern, showing a pronounced decrease between ages 10 and 20 [113,115]. The age trajectory of superior frontal cortical thickness in healthy individuals follows a similar pattern. After a rapid decrease until around age 20, cortical thickness stagnates before declining again after 30. In acAN patients, this trajectory appears systematically shifted to lower thickness values. Our analysis revealed significant thinning of the superior frontal cortex in acAN compared to HC, with a medium effect size (*d* = −0.41, *p*_fdr_ = 0.031), independent of age. Interestingly, in wrAN, cortical thickness in this region appeared to normalize.

Given the negative SHAP values, our model links higher superior frontal cortical thickness to a younger brain age. Therefore, the observed thinning in this region may contribute to the elevated brain-PAD in acAN. Additionally, the normalization of superior frontal cortical thickness after weight recovery may help explain the normal brain-PAD in wrAN.

Although the overall brain-PAD indicates an advanced brain age in patients with acAN, it is important to note that the structural brain changes observed in acAN do not simply reflect an accelerated form of normal aging. Of the three most critical features used by our model to estimate brain age, only one was altered in a way that could explain the overestimation in acAN. Additionally, we found that brain-PAD was lower in older patients with longer disease duration. If the brain changes in acAN represented accelerated healthy aging, we would expect brain-PAD to increase with age. This suggests that the structural alterations in acAN are more complex than a mere accelerated version of normal aging.

### 4.4. Comparison with previous studies

To date, only one published study has examined brain age in individuals with AN [67]. While this pioneering work provided initial evidence of accelerated brain aging in adolescent patients, it was limited by several methodological constraints. Their model was trained on a relatively small sample of 226 female participants aged 5–23 years, using the relevance vector regression (RVR). Feature extraction was limited to two structural metrics, GM and WM, derived from voxel-based morphometry (VBM). Moreover, their test set covered a narrow age range (12–23 years), limiting generalizability.

Despite these limitations, the study benefited from a longitudinal design two-time points, focusing primarily on acAN and partial weight restoration. At baseline, acAN patients showed an elevated BrainAGE_GM_ of +1.79 years compared to HC (95% CI [1.45, 2.13]), which significantly decreased after partial weight gain (*β* = −1.69; CI [−1.93, −1.46]). Interestingly, BrainAGE did not correlate with symptom severity or depression, but greater weight gain predicted stronger normalization of BrainAGE_GM_ (*β* = −0.65; CI [−0.75, −0.54]).

In contrast, our study was designed to address many of these methodological limitations. We compiled a large, age-diverse training dataset of 3487 healthy controls (HCs) across a broad age range (5–45 years), drawn from nine public databases and the JUH cohort. Our test set included 113 acAN, 35 wrAN, and age-matched HCs from both JUH and MGH institutions (see Table 1), allowing us to evaluate brain aging across both adolescence and adulthood.

MRI data were processed using FreeSurfer to extract a comprehensive set of 377 cortical and subcortical morphometric features. These served as inputs to two machine learning models, SVR and DKL-GPR. The DKL-GPR model demonstrated superior performance, achieving an MAE of 1.93 years and a high correlation (*r* = 0.88) in the age-matched HC test set (*n* = 90).

Testing the model on acAN participants revealed significantly elevated brain-PAD value (mean = +2.25 years), indicating advanced brain aging. Notably, brain-PAD normalized in the wrAN group (+0.26 years), with significant differences observed between wrAN and acAN (*p* = 0.0026), but not between wrAN and HC (*p* = 0.98). In acAN, brain-PAD was significantly negatively correlated with BMI (*r* = −0.291, *p* = 0.005) and illness duration (r = −0.423, *p* = 0.0078), consistent with the hypothesis that lower BMI and shorter illness duration are associated with greater deviations from normative aging. Unlike the prior study, we also examined associations between brain-PAD and eight clinical scales, but no statistically significant correlations remained after FDR correction.

In addition to global brain age measures, we performed regional comparisons of brain structure between groups. Significant reductions in right inferior parietal volume, thickness, and left precentral volume were observed in acAN patients. SHAP analysis further revealed that left superior frontal thickness was significantly reduced in acAN, a finding partially reversed in wrAN participants, who showed increased superior frontal thickness. However, hippocampal volumes continued to decrease in wrAN participants, with a more prominent reduction in the right hippocampus (*d* = 0.31, n.s.) compared to the left (*d* = 0.12, n.s.) (see Fig. 5b and Fig. 5c).

Together, these findings highlight the advantages of our approach, which not only addresses the limitations of prior work but also provides deeper insights into the structural brain alterations associated with AN.

### 4.5. Limitations of the study

While this study offers important insights into advanced brain aging in individuals with AN, several limitations should be acknowledged. First, the sample size for the wrAN group was relatively small, which may have reduced statistical power and limited the generalizability of findings regarding structural normalization following weight recovery. Second, although participants in the wrAN group met clinical recovery criteria, we lacked consistent data on the duration of weight restoration prior to MRI acquisition, which prevented us from analyzing the potential impact of time at a healthy weight on brain structure. Future studies should aim to capture longitudinal weight recovery data to more precisely model the dynamics of structural normalization. Third, our study included preadolescent and adolescent participants, raising concerns about segmentation accuracy given that FreeSurfer’s cortical and subcortical atlases were developed using adult brain templates. We acknowledge this concern and emphasize that previous studies have demonstrated the feasibility and acceptable accuracy of FreeSurfer in pediatric populations, including children as young as five years old [116–120]. Furthermore, we conducted extensive visual quality assessments, particularly for younger participants, and confirmed that the segmentations were of satisfactory quality. Notably, the observed developmental patterns in key cortical regions, such as the superior frontal cortex, were consistent with established normative trajectories across the lifespan [46,121], further supporting the validity of our findings in younger cohorts.

Fourth, sample heterogeneity, in terms of illness duration, severity, comorbid conditions, and treatment history, may have introduced additional variability that was not fully accounted for in our analysis. Future studies with more detailed clinical metadata could help disentangle these factors. Lastly, the cross-sectional design of the current study precludes direct inference about the trajectory of brain aging over time. Longitudinal follow-up will be critical to determine whether brain-PAD changes observed in acute AN persist, reverse, or fluctuate with treatment and sustained recovery. By acknowledging these limitations, we aim to guide future research efforts toward more refined, longitudinal, and developmentally sensitive investigations of brain aging in AN populations.

Additionally, although the test groups were unbalanced in size, we used statistical methods (GLMs with robust post-hoc correction) that are well-suited for such designs, and all group comparisons were interpreted with this limitation in mind.

## 5. Conclusions

This study extends brain age research in anorexia nervosa by applying a high-performance deep kernel learning model trained on a large, age-diverse female sample of healthy controls (ages 5–45) and tested on female patients across a wide clinical age range (10–40 years). By integrating a hybrid DKL-GPR model and an expanded neuroanatomical feature set, we achieved improved prediction accuracy and interpreability over conventional approaches.

Our results demonstrate that patients with acute anorexia nervosa (acAN) exhibit significantly elevated brain-PAD values, suggesting older-appearing brains relative to their chronological age. This overestimation was most prominent in individuals with lower BMI and shorter illness duration. In contrast, brain-PAD in weight-restored AN (wrAN) patients did not differ from healthy controls, and BMI was not a significant predictor of brain-PAD in this group. These findings suggest that advanced brain aging in acAN may be more closely tied to acute malnutrition than to irreversible neurodegenerative processes.

Importantly, among the model’s most influential brain regions, only the superior frontal cortex exhibited structural changes consistent with elevated brain-PAD in acAN. This implies that advanced brain aging in AN may not represent a generalized acceleration of normative aging, but rather reflects a distinct and regionally specific neurobiological pattern.

Together, these findings underscore the value of brain age prediction models for identifying subtle but clinically meaningful deviations in brain structure in eating disorders. Future longitudinal studies are needed to clarify the temporal dynamics of brain-PAD changes, their relationship to treatment response, and the potential for structural recovery across different illness stages.

## Supplementary Material

1

2

3

4

5

6

7

## Figures and Tables

**Table 1:** Demographic variables and clinical measures from two sites (JUH and MGH).

General	Jena University Hospital (JUH)		Massachusetts General Hospital (MGH)		
	HC	acAN	HC	acAN	wrAN
Nos. of patients	42	42	48	71	35

Age mean (SD)	23.8 ± 3.7	23.5 ± 5.2	18.8 ± 3.3	19.9 ± 3.9	22.7 ± 3.9
Age range	18.6 – 37.8	18.2 – 40.0	11.1 – 24.0	10.1 – 32.7	16.3 – 34.9
BMI (kg/m^2^)	23.3 ± 3.4	15.7 ± 1.7	21.5 ± 2.0	16.8 ± 1.34	20.9 ± 1.6

Mean ± Standard deviation (SD) values for each variable. HC, healthy control subjects; acAN, acute anorexia nervosa patients; wrAN, weight recovered anorexia patients; BMI, body mass index.

**Table 2:** Clinical measures of acAN and age-matched HC testing set from JUH and their group differences.

Clinical scales	HC			acAN			Comparison between HC and acAN		
	M ± SD	*r*	*p*_fdr_	M ± SD	*r*	*p*_fdr_	*t*	*df*	*p*

TMTa	26 ± 7.8	0.03	0.87	23.6 ± 8.4	−0.16	0.61	1.39	80.23	0.17
TMTb	49.7 ± 16	−0.09	0.75	51.3 ± 16.1	−0.16	0.61	−0.44	80.9	0.65
BIS	56.5 ± 7.9	−0.3	0.75	58.6 ± 11.7	0.25	0.52	−0.91	68.21	0.36
TAS26	40.9 ± 9.3	0.11	0.75	52.1 ± 10.1	−0.11	0.71	−4.69	63.85	<.001
EDI-2	209.4 ± 40.7	0.06	0.75	310 ± 45.5	−0.21	0.57	−10.16	70.90	<.001
BDI-II	5.7 ± 5.8	−0.09	0.75	25.5 ± 11.1	−0.17	0.69	−9.67	61.43	<.001
STAI-S	33.8 ± 5.8	−0.09	0.75	46.7 ± 9.8	0.04	0.83	−7.37	67.09	<.001
STAI-T	43.4 ± 6.2	0.21	0.75	48.8 ± 8.1	−0.04	0.83	−3.40	72.78	0.001
BMI admission (kg/m^2^)	.	.	.	15.2 ± 1.6	−0.27	0.52	.	.	.
BMI discharge (kg/m^2^)	.	.	.	16.1 ± 1.5	−0.06	0.83	.	.	.
Age of onset (years)	.	.	.	16.9 ± 3.6	0.14	0.61	.	.	.

Mean ± Standard deviation (SD) values for each variable. HC, healthy control subjects; acAN, acute anorexia nervosa patients; EDI-2, Eating Disorder Inventory-2; BDI-II, Beck Depression Inventory-II; TMTa, Trail Making Task - A; TMTb, Trail Making Task - B; BIS, Behavioral Inhibition System; STAI-S, State Anxiety Inventory; STAI-T, Trait Anxiety Inventory. The mean duration of illness was 6.15 ± 5.75 (SD) years. BMI admission and BMI discharge are the body mass index assessed at admission and discharge from the hospital.

## Data Availability

Most of the data used in this study are publicly available and can be accessed directly from their webpages, except for the data provided by JUH, whereas some of the MGH data can be available through https://nda.nih.gov/ or upon request to the principal investigators. JUH data cannot be shared due to confidentiality agreements required by the JUH institutional review board (IRB). However, the trained brain age prediction model and all code necessary to reproduce the main results are available at: https://github.com/Yuvi-416/AN-BrainAGE.

## Appendix A. Supplementary data

The following is/are the supplementary data to this article: Supplementary material file 1: HC Data Source Excel sheet; Supplementary material file 2: Extended Manuscript Text; Supplementary material file 3: Group-level Comparison Excel sheet.

Supplementary material related to this article can be found online at https://doi.org/10.1016/j.compbiomed.2025.110484.
