# Supplementary material for: Does restrictive anorexia nervosa impact brain aging? A machine learning approach to estimate age based on brain structure
Source: Comput Biol Med. Author manuscript; Available in PMC 2026 Jul 20. (PMC13382908; doi:10.1016/j.compbiomed.2025.110484)
Supplement: 1 [file NIHMS2177580-supplement-1.pdf]

Does Restrictive Anorexia Nervosa Impact Brain Aging? A Machine Learning Approach to Estimate Age Based on Brain Structure

Yubraj Gupta[a,*], Feliberto de la Cruz[a,b], Katrin Rieger[a], Monica di Giuliano[a], Christian Gaser[c,d,o], James Cole[e,f], Lauren Breithaupt[g,h,i], Laura M. Holsen[b,i,k], Kamryn T. Eddy[g,h,i], Jennifer J. Thomas[g,h,i], Suheyla Cetin-Karayumak[b,h], Marek Kubicki[b,h,j], Elizabeth A. Lawson[i,k,l], Karen K. Miller[k,l], Madhusmita Misra[i,l,m,n], Andy Schumann[a,**], Karl-Jürgen Bär[a,**]

[a]*Department of Psychosomatic Medicine and Psychotherapy, Jena University Hospital, Jena, Germany*
[b]*Department of Psychiatry, Brigham and Women's Hospital, Harvard Medical School, Boston, USA*
[c]*Department of Psychiatry and Psychotherapy, Jena University Hospital, Jena, Germany*
[d]*Department of Neurology, Jena University Hospital, Jena, Germany*
[e]*Centre for Medical Imaging Computer, University College London, London, UK*
[f]*Dementia Research Centre, University College London, London, UK*
[g]*Eating Disorders Clinical and Research Program, Massachusetts General Hospital, Boston, USA*
[h]*Department of Psychiatry, Harvard Medical School, Boston, USA*
[i]*Mass General Brigham Multidisciplinary Eating Disorder Research Collaborative, Massachusetts General Hospital, Boston, USA*
[j]*Athinoula A. Martinos Center, Massachusetts General Hospital, Boston, USA*
[k]*Harvard Medical School, Boston, USA*
[l]*Neuroendocrine Unit, Massachusetts General Hospital, Boston, USA*
[m]*Division of Pediatric Endocrinology, University of Virginia, Charlottesville, USA*
[n]*Department of Pediatrics, University of Virginia, Charlottesville, USA*
[o]*German Center for Mental Health (DZPG), Germany*

1. Machine Learning Algorithms

1.1. SVR Implementation

The SVR model [1] that we are implementing aims to find the function given below:

$$f(x) = u^T \phi(x) + b, \tag{1}$$

The goal is to predict the target variable as accurately as possible for each input vector $x \in \mathbb{R}^p$, where:

- x is a p-dimensional feature vector corresponding to the neuroimaging data for a single subject.

*Corresponding author: Yubraj.Gupta@med.uni-jena.de
**Shared senior-authors.

- $\phi(x)$ represents the high-dimensional space to which x is mapped using the radial basic function (RBF) kernel. This mapping allows linear regression in the transformed feature space, capturing non-linear relationships in the original input space.

- $u \in \mathbb{R}^p$ is the weight vector in the transformed feature space, determining the orientation and magnitude of the hyperplane used for regression.

- $b \in \mathbb{R}$ is the bias term, adjusting the offset of the regression hyperplane in the high-dimensional space.

RBF Kernel, defined as:

$$K(x_i, x_j) = exp(-\gamma \left\| x_i - x_j \right\|^2), \tag{2}$$

Where γ is a parameter controlling the width of the kernel and facilitates the transformation of the input space into a higher-dimensional space. This kernel is particularly suited for handling the complex, non-linear patterns often present in neuroimaging data.

So, the objective of the SVR is to optimize the function:

$$\min_{u,\xi,\xi^*} \frac{1}{2} \|u\|^2 + c \sum_{i=1}^{n} (\xi_i + \xi_i^*), \tag{3}$$

Subject to the constraints:

$$\begin{gathered} y_i - (u^T \phi(x_i) + b) \leq \varepsilon + \xi_i, \\ (u^T \phi(x_i) + b) - y_i \leq \varepsilon + \xi_i^*, \\ \xi_i, \xi_i^* \geq 0 \end{gathered}$$

Where:

- y_i is the actual target value for the ith sample.

- ϵ specifies the margin of tolerance within which no penalty is given for errors.

- C is the regularization parameter, which balances the trade-off between the smoothness of the regression function and the amount up to which deviations larger than ϵ are tolerated.

- ξ_i and ξ_i^* are slack variables that allow deviations larger than ϵ for below and above the predicted values, respectively.

1.2. GPR and DKL-GPR Implementation

A Gaussian process (GP) [2] is a collection of random variables, any finite number with a joint Gaussian distribution. In the context of regression, it is used to define a prior over function $f(x)$, which is provided below:

$$f(x) \sim GP(\mu(x), k(x, x'; \gamma)), \tag{4}$$

$$y = f(x) + \varepsilon,$$

$$\varepsilon \sim \mathcal{N}(0, \sigma_\varepsilon^2),$$

Where the GP is characterized by its mean function $\mu(x) = \mathbb{E}[f(x))]$ and positive definite covariance (kernel) function $k(x, x'; \gamma) = k_\gamma(x, x') = \mathbb{E}[(f(x) - \mu(x))(f(x') - \mu(x'))]$ hyperparameter γ, x and x' being two possible pairs in the input domain, the mean function can often be taken as zero without losing generality. The covariance function (kernel) encodes assumptions about the function which we want to learn. Similarly, ε is an independent added Gaussian noise term with variance σ_ε^2. The popular choice of the kernel is the RBF kernel due to its properties of being infinitely differentiable and capturing varying degrees of similarity between points, and it is defined as:

$$k_\gamma(x, x') = \sigma_f^2 exp(-\frac{||x - x'||^2}{2l^2}), \tag{5}$$

Where, $||x - x'||^2$ is the squared Euclidean distance between the feature vectors x and x', l is the length-scale parameter, which determines how quickly the correlation between two points decreases with distance, and σ_f^2 is the variance parameter, controlling the overall variance of the functions drawn from the GP.

Given a set of training data (X, Y) where $X \epsilon \mathbb{R}^{m*n}$ represents the feature matrix and $Y \epsilon \mathbb{R}^n$ is the vector of observed target values, GPR aims to predict the target values Y^* for a new set of input features X^*. The predictive distribution of Y^* given X^*, X, and Y is also Gaussian, which can be shown by the below equation:

$$Y^*|X^*, X, Y \sim \mathcal{N}(\mu^*, \Sigma^*), \tag{6}$$

where the mean μ^* and covariance Σ^* of the predictive distribution are given by:

$$\mu^* = k_\gamma(X^*, X)[k_\gamma(X, X) + \sigma_\varepsilon^2 I]^{-1} Y, \tag{7}$$

$$\Sigma^* = k_\gamma(X^*, X^*) - k_\gamma(X^*, X)[k_\gamma(X, X) + \sigma_\varepsilon^2 I]^{-1} K(X, X^*) \tag{8}$$

Where $k_\gamma(X, X)$ is the covariance matrix computed between all pairs of training inputs X using the RBF kernel, $k_\gamma(X^*, X)$ is the covariance matrix computed between the test inputs X^* and the training inputs X, $k_\gamma(X^*, X^*)$ is the covariance matrix computed between all pairs of test inputs X^*, σ_ε^2 is the noise variance, representing the variance of the observational noise in the target values Y, and I is the identity matrix of appropriate size.

GP regression is a popular probabilistic ML model for regression and uncertainty estimation. However, its scalability is limited when dealing with high-dimensional inputs, often called the curse of dimensionality. To mitigate this issue, deep kernel learning (DKL) [3] was utilized to leverage the non-linear expressive power of DNN to learn compressed data representations while still retaining the essential probabilistic features of kernel-based GP model for accurately quantifying uncertainty.

The primary goal of DKL-GPR is to transform high-dimensional input features into a lower-dimensional, more informative feature space using a DNN. After feature transformation, GPR is applied to the transformed features. The fundamental idea of GPR is to impose a GP prior over functions, which is fully specified by its mean function μ^* (often set to zero) and its covariance function or kernel Σ^*, which specifies the covariance between any two points in the transformed feature space.

In essence, DKL embeds a DNN, representing a non-linear mapping from the data to the feature space, into the kernel function for GPR. Specifically, the kernel function can be expressed as follows:

$$k_{DKL}(x, x'; \gamma, \theta) = k_\gamma(g(x; \theta), g(x'; \theta)), \tag{9}$$

Here, $g(x; \theta)$ represents a non-linear mapping given by deep architecture, such as a deep convolutional network, with input x and parameters (weight and biases) θ. This approach enables us to learn more informative representations of high-dimensional data, which can significantly improve the performance of GP regression in practice.

2. Group-Level Neuroanatomical Comparisons

In the supplementary material, Figure S1 presents additional group-level comparisons of neuroanatomical changes between acutely underweight anorexia nervosa (acAN) patients and healthy controls (HC) from the JUH dataset. These comparisons highlight widespread cortical and white matter alterations, particularly in regions associated with cognitive and motor functions. Notably, cortical thickness and volume reductions are observed in the frontal and parietal lobes, which are consistent with the neuroanatomical impacts of malnutrition. These changes align with the cortical alterations seen in the broader dataset analysis shown in Figure 5a of the main manuscript. The additional focus on JUH data in Figure S1 reinforces the generalizability of these findings across different cohorts, demonstrating that the structural reductions in cortical regions observed in acAN are a consistent marker of the disorder.

Figure S2 further expands on the analysis of subcortical structures, comparing acAN patients to HC and wrAN groups. The figure illustrates how subcortical regions like the thalamus and hippocampus are particularly affected in acAN, with notable reductions in volume. When weight-restored anorexia nervosa patients (wrAN) are included, the results show partial recovery in certain regions, though persistent deficits remain, especially in the hippocampus. These findings are consistent with the main manuscript's observations of structural recovery in some brain regions following weight restoration (Figure 5b). However, they also highlight that certain neuroanatomical changes may not fully normalize with weight gain. This further reinforces the need for early and sustained interventions to mitigate long-term brain aging in AN patients.

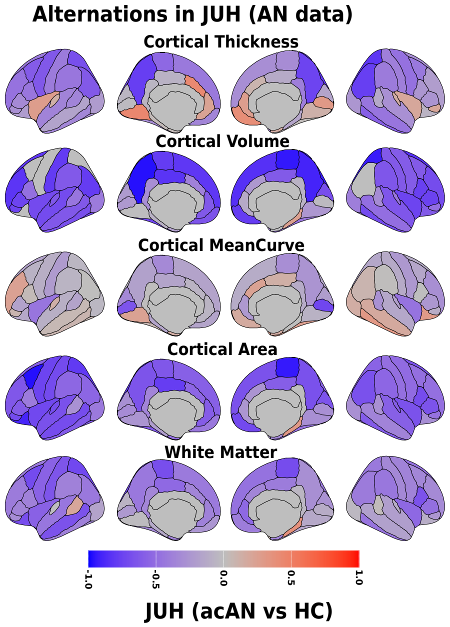

Figure 1: Group-level neuroanatomical comparisons between acutely underweight anorexia nervosa (acAN) patients and healthy controls (HC) from the JUH dataset. The figure illustrates alterations in cortical and white matter structures across five measures: cortical thickness, cortical volume, cortical mean curvature, cortical surface area, and white matter volume. Group comparisons were computed using Cohen's *d* effect size, with negative values (blue) indicating reductions in acAN relative to HC and positive values (red) representing increases. Significant reductions in acAN patients are mainly observed in cortical thickness and volume, with notable effects in frontal and parietal regions, consistent with the neuroanatomical impact of malnutrition on brain structure. White matter differences are also evident, further reflecting widespread structural changes associated with AN. The color bar denotes the Cohen's *d* effect size, ranging from -1.0 (blue) to +1.0 (red), with zero (gray) indicating no group differences.

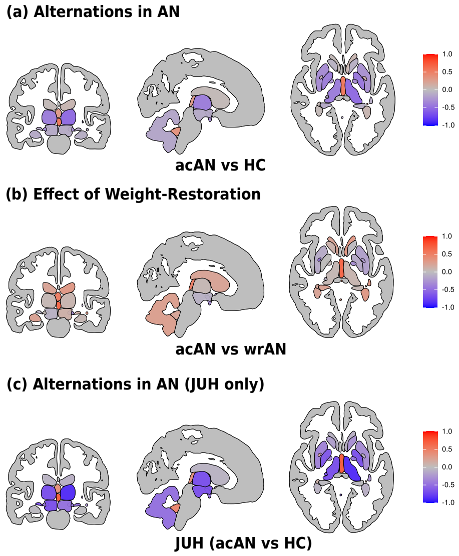

Figure 2: Subcortical Neuroanatomical Alterations in AN and wrAN Patients. This figure illustrates group-level subcortical brain structure comparisons for (a) acAN vs. HC, (b) acAN vs. wrAN, and (c) JUH acAN vs. HC, using Cohen's d effect sizes. Subcortical structures, including the thalamus, hippocampus, and amygdala, are visualized across multiple views. In panel (a), acAN patients show significant volume reductions compared to HC, particularly in the thalamus and hippocampal regions, consistent with accelerated brain aging and malnutrition-related structural changes. Panel (b) highlights the impact of weight restoration in wrAN, revealing partial recovery in some subcortical regions, such as the thalamus, though certain deficits persist, such as in the hippocampus. Panel (c) presents findings from the JUH cohort, where the acAN group demonstrates notable reductions in subcortical volumes compared to HC, consistent with the results seen in the primary cohort. The color bar represents Cohen's d effect sizes, ranging from -1.0 (blue), indicating volume reductions in AN relative to HC or wrAN, to +1.0 (red), indicating volume increases.

The supplementary material integrates Figure S1 and S2 with the primary analyses and underscores the robustness of these neuroanatomical changes across different datasets. The consistency between the JUH-specific findings in the supplementary material and the combined dataset results in the main manuscript lends additional support to the conclusion that accelerated brain aging in anorexia nervosa is a widespread and significant phenomenon.

3. Brain-PAD in HC, acAN, and wrAN Patients across Validation, JUH, and MGH Datasets

This figure presents an analysis of brain-PAD values across HC, acAN, and wrAN patients from multiple sources (validation set, JUH, MGH). Panel (a) illustrates the relationship between chronological age and brain-PAD in the HC test group (p = 90). The weak and non-significant correlation (r = 0.178, p = 0.093) indicates that the model's predictions are not confounded by age within the test sample, supporting the robustness of brain age estimates across the examined age range.

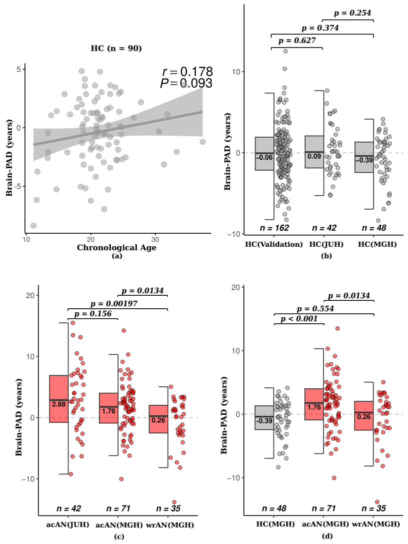

Figure 3: Brain-PAD in HC, acAN, and wrAN Participants Across Validation, JUH, and MGH Datasets. (a) Scatterplot showing the relationship between brain-PAD and chronological age in the HC test group (n = 90). No significant age dependency was found ($r = 0.178$, $p = 0.093$), supporting the model's generalizability across the test age range. (b) Brain-PAD distributions in healthy controls from the validation set (n = 162), JUH site (n = 42), and MGH site (n = 48), with no significant differences between subgroups (all $p > 0.25$), confirming consistency of normative aging across datasets. (c) Brain-PAD values in acAN patients from JUH (n = 42) and MGH (n = 71), as well as wrAN patients from MGH (n = 35). acAN participants showed significantly elevated brain-PAD compared to both HC and wrAN ($p < 0.001$ and $p = 0.0134$, respectively), while wrAN patients showed partially normalized brain age. (d) Group comparison within the MGH dataset confirms a significant difference between acAN and HC ($p < 0.001$), and between acAN and wrAN (p = 0.0134), but no difference between HC and wrAN ($p = 0.554$), suggesting recovery-related improvements in brain age.

Panel (b) compares brain-PAD values across healthy control groups from the validation dataset (n = 162), JUH (n = 42), and MGH (n = 48). No significant differences were observed between these subgroups (all $p > 0.25$), demonstrating the consistency of brain age predictions among healthy individuals across different datasets. Panel (c) shows brain-PAD values for acAN participants from JUH (n = 42) and MGH (n = 71), as well as wrAN participants from MGH (n = 35). Both acAN groups exhibit elevated brain-PAD values, with JUH patients showing a higher median brain-PAD (+2.88 years) than those from MGH (+1.76 years). While the difference between JUH and MGH acAN groups was not statistically significant ($p = 0.156$), both groups significantly differed from the wrAN group ($p = 0.0134$ and $p = 0.00197$, respectively), reinforcing the presence of advanced brain aging in the acute stage of AN and highlighting the potential effects of weight restoration. Panel (d) provides within-site comparisons at MGH, where acAN participants show significantly higher brain-PAD than

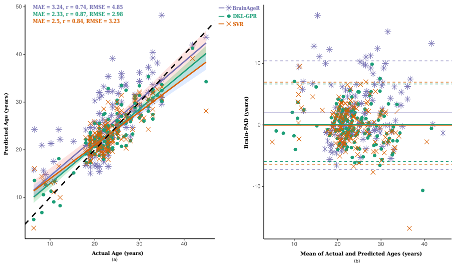

Figure 4: Comparative Performance of Brain Age Prediction Models (DKL-GPR, SVR, and BrainAgeR) on the Testing Set. (a) A scatter plot compares predicted brain age with actual chronological age for DKL-GPR, SVR, and BrainAgeR models. The DKL-GPR model demonstrated the highest correlation (r = 0.87), lowest MAE = 2.33, and lowest RMSE = 2.98, outperforming both SVR (r = 0.84, MAE = 2.5) and BrainAgeR (r = 0.74, MAE = 3.24). (b) The Bland-Altman plot shows brain-PAD values across the models with their bias and upper and lower limits. The DKL-GPR model shows minimal bias (+ 0.066) with the narrowest 95% limits of agreement (-5.79 to +5.92 years), indicating its higher accuracy and reliability in estimating brain age than the other models.

both HC ($p < 0.001$) and wrAN ($p = 0.0134$). The wrAN group does not differ significantly from HC ($p = 0.554$), indicating that weight restoration is associated with normalization of brain age.

This figure underscores the replicability of advanced brain aging in acAN across sites and its partial reversal following weight restoration. The consistent results across multiple datasets highlight the robustness of the observed effects and the potential utility of brain-PAD as a biomarker for neurobiological recovery in AN.

References

[1] A. J. Smola, B. Schölkopf, A tutorial on support vector regression, Statistics and computing 14 (2004) 199–222.

[2] C. Rasmussen, C. Williams, Gaussian processes for machine learning.,(mit press: Cambridge, ma) (2006).

[3] A. G. Wilson, Z. Hu, R. Salakhutdinov, E. P. Xing, Deep kernel learning, in: Artificial intelligence and statistics, PMLR, 2016, pp. 370–378.
