# Supplementary material for: Does restrictive anorexia nervosa impact brain aging? A machine learning approach to estimate age based on brain structure
Source: Comput Biol Med. Author manuscript; Available in PMC 2026 Jul 20. (PMC13382908; doi:10.1016/j.compbiomed.2025.110484)
Supplement: 2 [file NIHMS2177580-supplement-2.pdf]

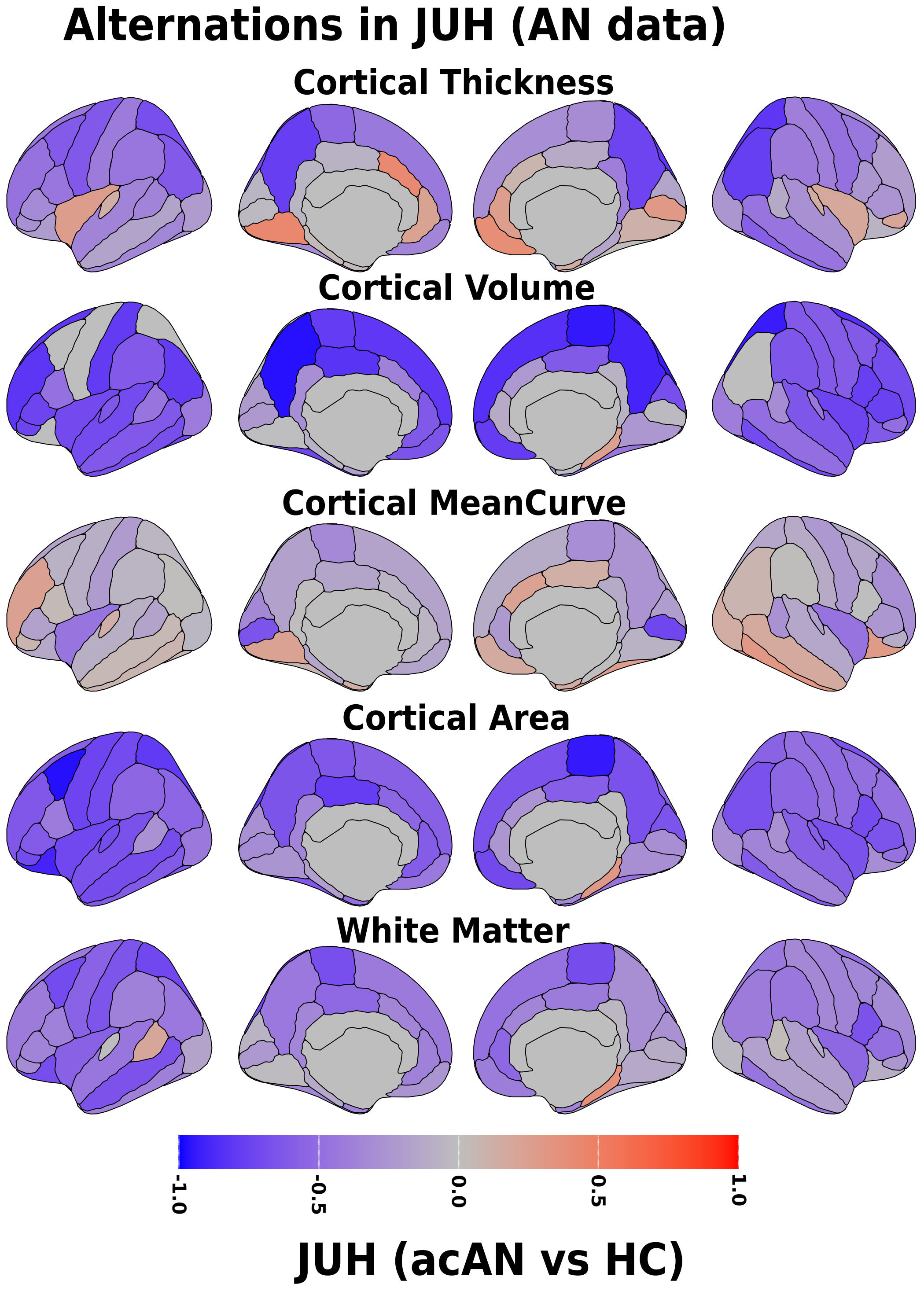

Alternations in JUH (AN data)
Cortical Thickness
Cortical Volume
Cortical MeanCurve
Cortical Area
White Matter
-1.0
-0.5
0.0
0.5
1.0
JUH (acAN vs HC)
