# Supplementary figures and images for: Does restrictive anorexia nervosa impact brain aging? A machine learning approach to estimate age based on brain structure
Source: Comput Biol Med. Author manuscript; Available in PMC 2026 Jul 20. (PMC13382908; doi:10.1016/j.compbiomed.2025.110484)

**(a) Alternations in AN**

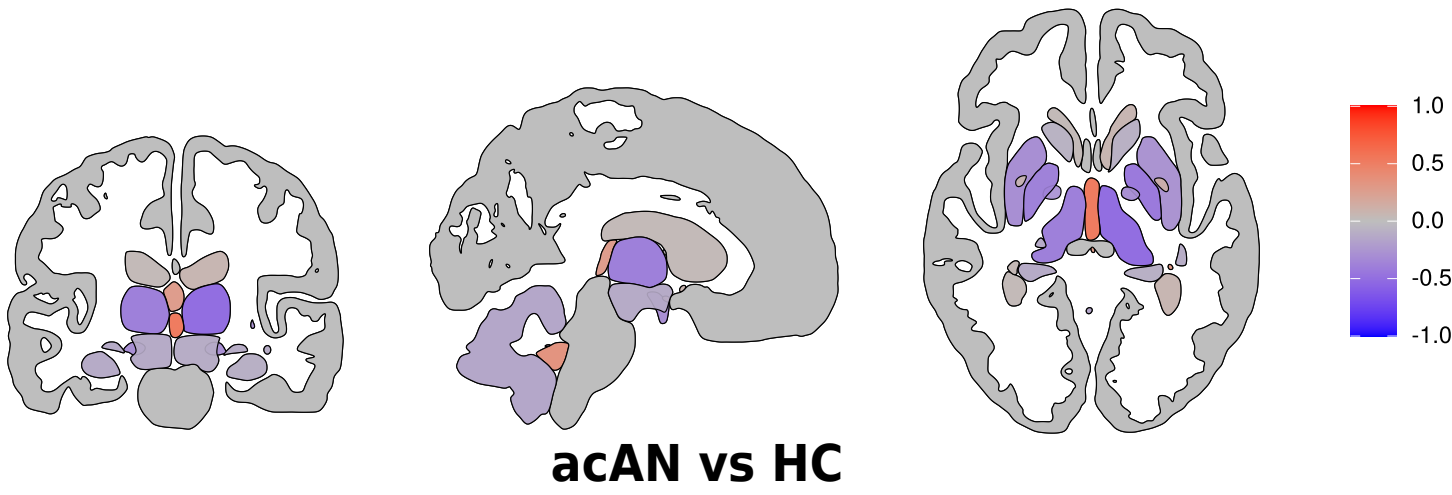

**(b) Effect of Weight-Restoration**

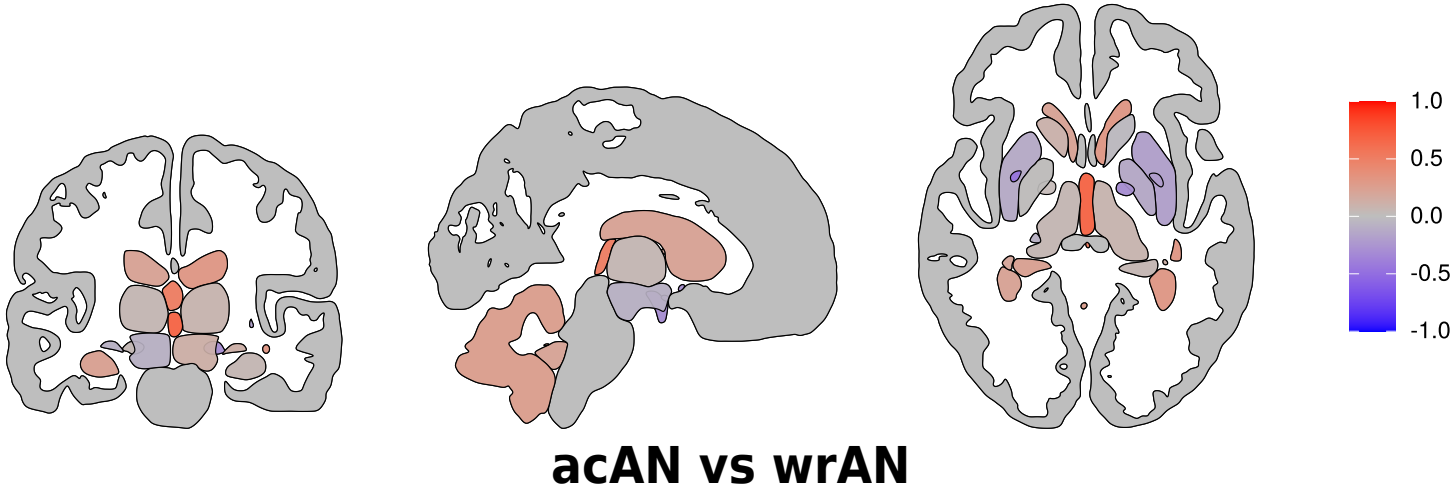

**(c) Alternations in AN (JUH only)**

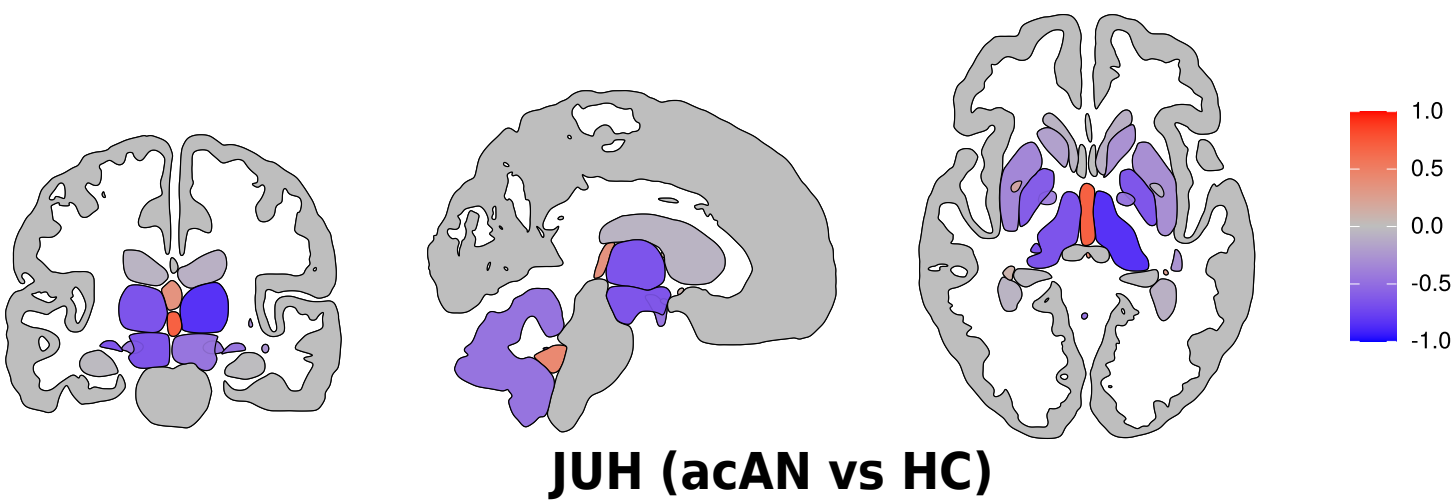

Supplement: 3 [file NIHMS2177580-supplement-3.pdf]

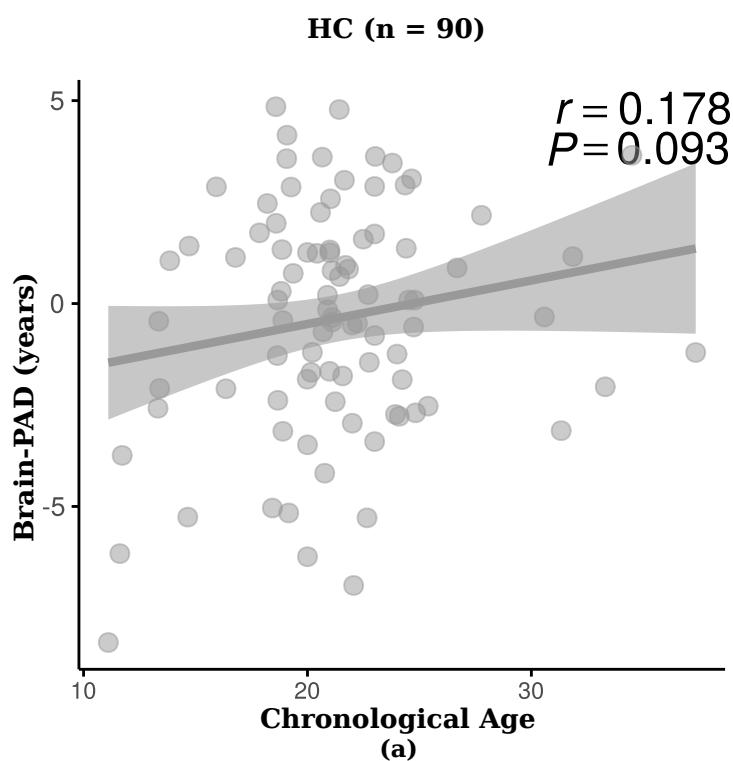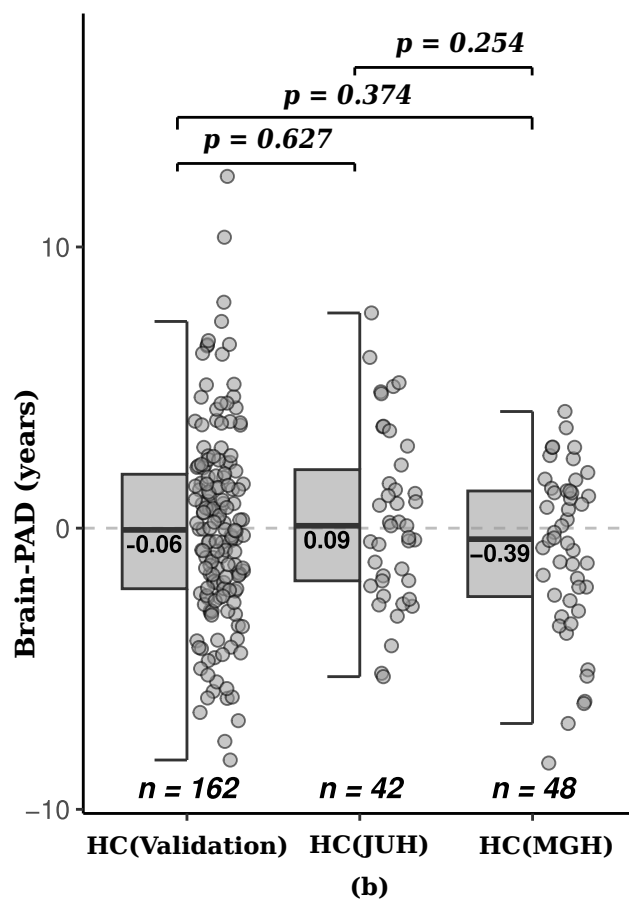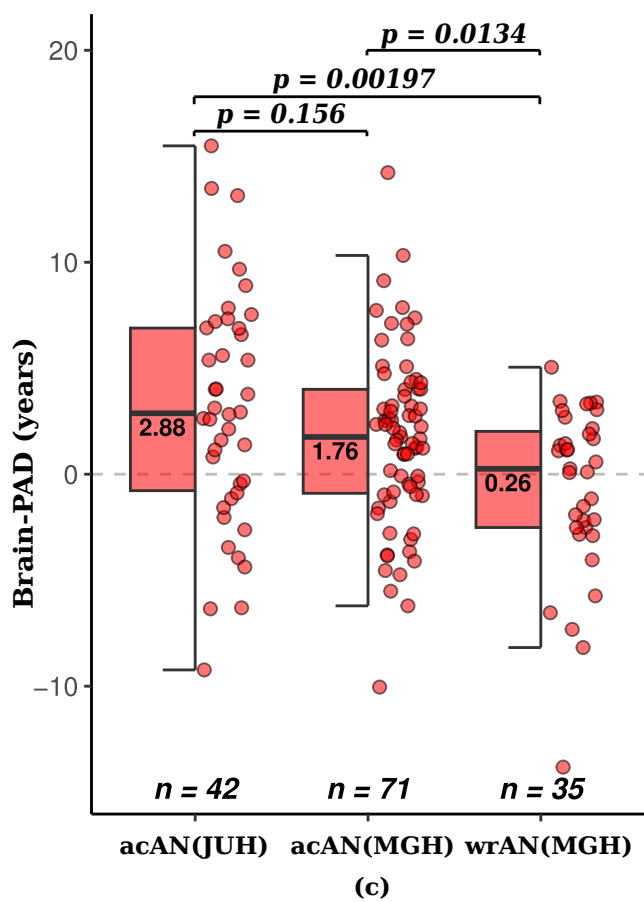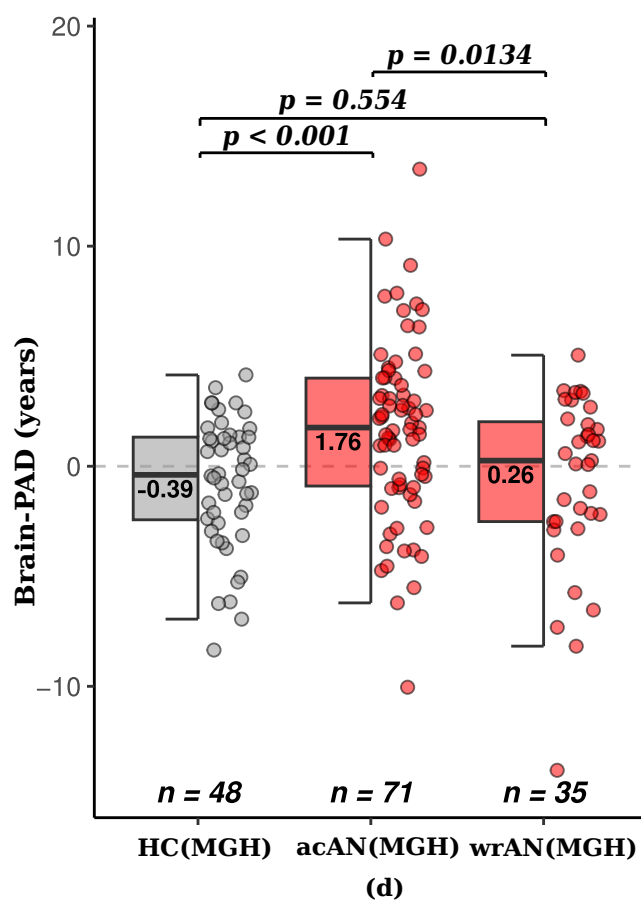

Supplement: 4 [file NIHMS2177580-supplement-4.pdf]

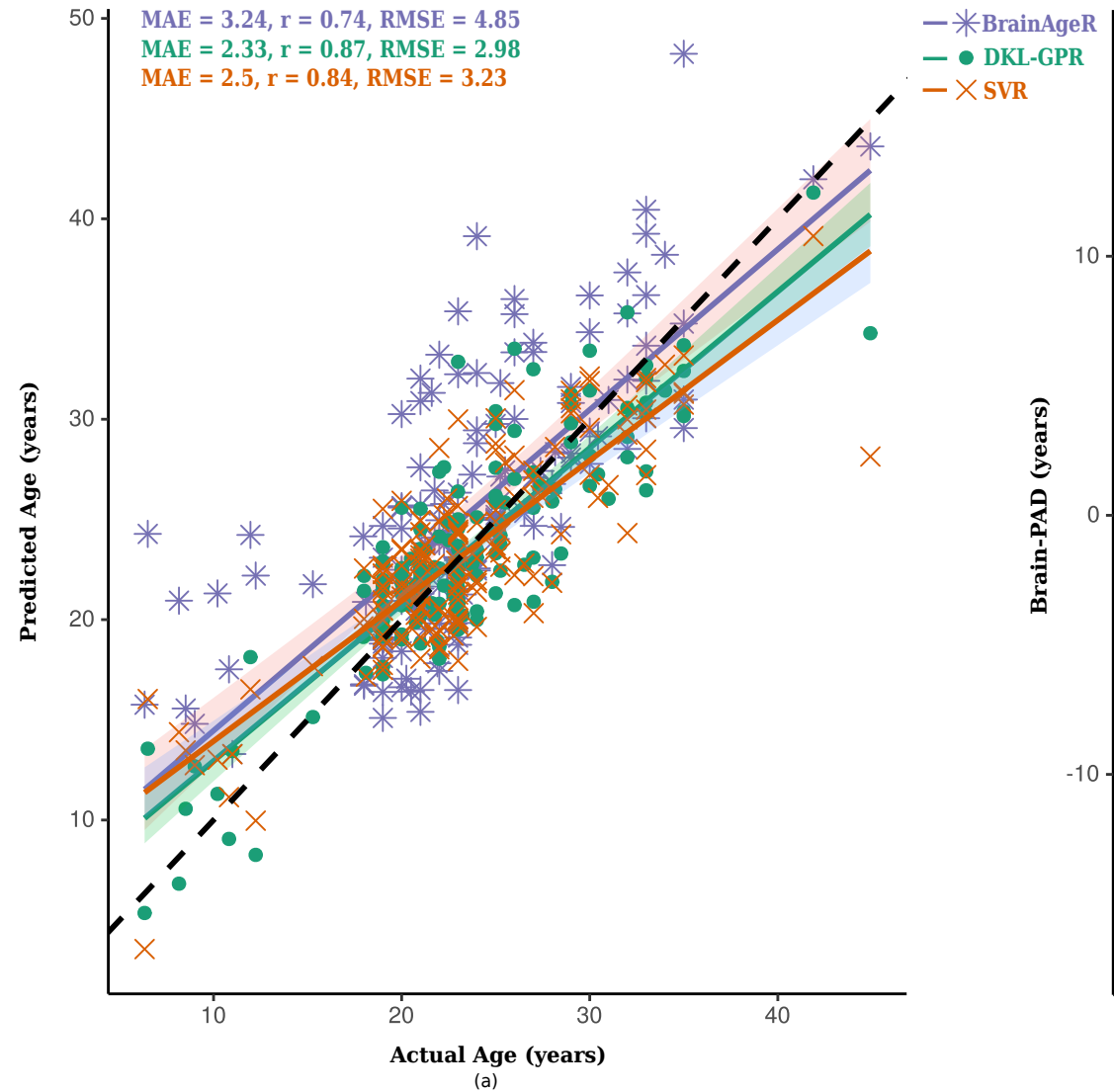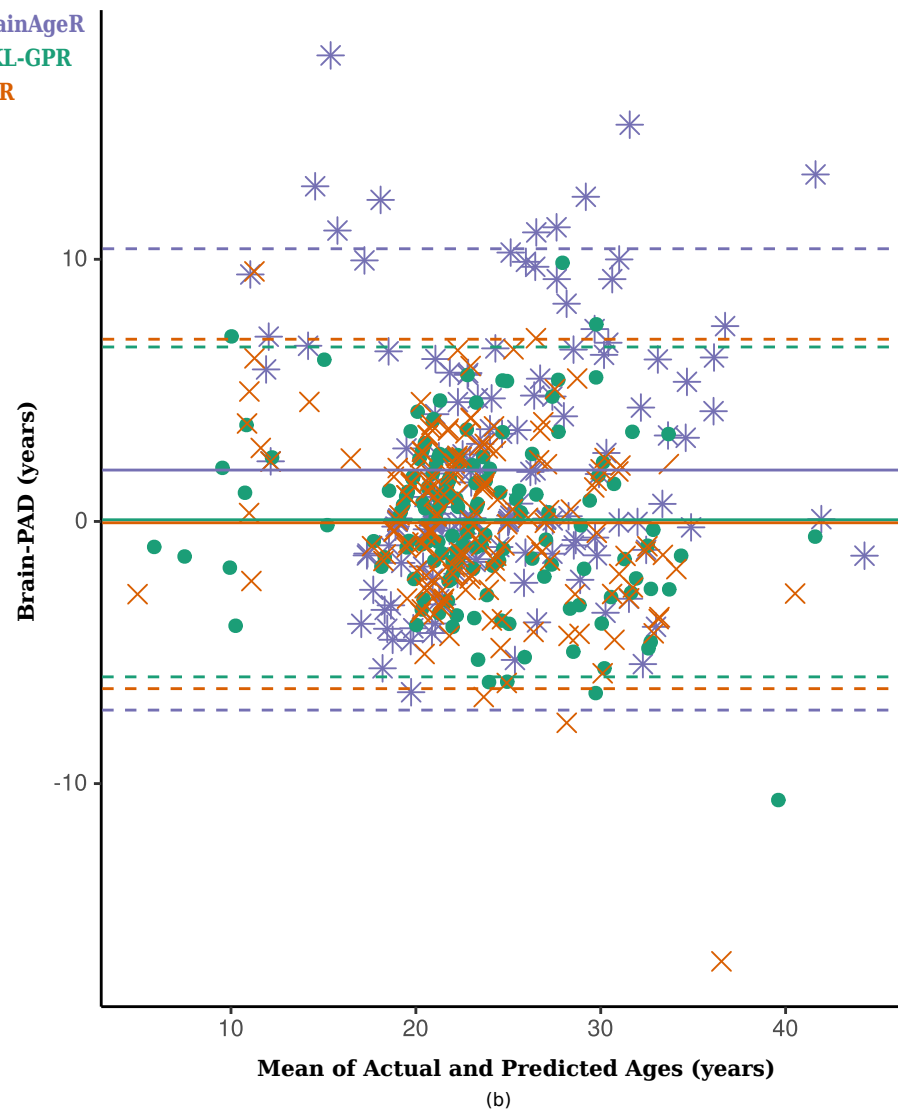

Supplement: 5 [file NIHMS2177580-supplement-5.pdf]
